# Gradual Change between Coherent and Incoherent Tunneling Regimes Induced by Polarizable Halide Substituents in Molecular Tunnel Junctions

**DOI:** 10.1021/jacs.4c06295

**Published:** 2024-08-08

**Authors:** Xiaoping Chen, Ira Volkova, Yulong Wang, Ziyu Zhang, Christian A. Nijhuis

**Affiliations:** † College of Chemistry, Chemical Engineering and Environment, Fujian Provincial Key Laboratory of Modern Analytical Science and Separation Technology, Minnan Normal University, Zhangzhou 363000, China; ‡ Department of Chemistry, National University of Singapore, 3 Science Drive 3, 117543 Singapore; § Centre for Advanced 2D Materials and Graphene Research Centre, National University of Singapore, 6 Science Drive 2, 117546 Singapore; ∥ Hybrid Materials for Optoelectronics Group, Department of Molecules and Materials, MESA+ Institute for Nanotechnology and Molecules Centre, Faculty of Science and Technology, University of Twente, 7500 AE Enschede, The Netherlands

## Abstract

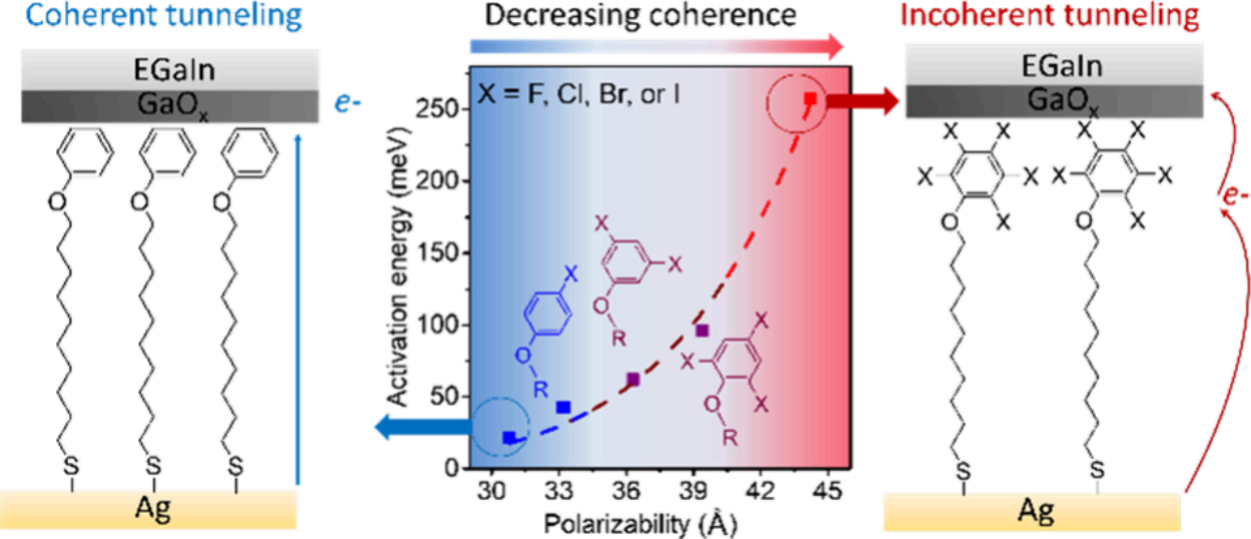

This paper describes a gradual transition of charge transport across molecular junctions from coherent to incoherent tunneling by increasing the number and polarizability of halide substituents of phenyl-terminated aliphatic monolayers of the form S(CH_2_)_10_OPhX_*n*_, X = F, Cl, Br, or I; *n* = 0, 1, 2, 3, or 5. In contrast to earlier work where incoherent tunneling was induced by introducing redox-active groups or increasing the molecular length, we show that increasing the polarizability, while keeping the organization of the monolayer structure unaltered, results in a gradual change in the mechanism of tunneling of charge carriers where the activation energy increased from 23 meV for *n* = 0 (associated with coherent tunneling) to 257 meV for *n* = 5 with X = Br (associated with incoherent tunneling). Interestingly, this increase in incoherent tunneling rate with polarizability resulted in an improved molecular diode performance. Our findings suggest an avenue to improve the electronic function of molecular devices by introducing polarizable atoms.

## Introduction

Understanding the underlying mechanisms of charge transport across molecules is important in countless areas of research ranging from interface engineering,^1−3^ catalysis,^4^ nanoelectronics,^5−8^ or energy,^9^ yet some intriguing phenomena still cannot be explained, such as long-range tunneling^10,11^ or chirality-induced spin selectivity (CISS).^12−14^ Molecular tunnel junctions of the form metal–molecule–metal where the molecule is present as monolayer (large area junctions), or single molecule, make it possible to study the mechanisms of charge transport with exquisite detail.^9,15−19^ Mostly, such junctions are assumed to operate in the coherent tunneling regime which is independent of temperature^20^ or in the incoherent tunneling regime (also called hopping) when charge transport is thermally activated.^18,21,22^ A matter of ongoing debate is when a junction switches between coherent and incoherent tunneling regimes, and when this transition is gradual^23^ or abrupt.^24−27^ Here we show that it is possible to access an ill-defined transition regime between coherent and incoherent tunneling by introducing soft, polarizable atoms gradually changing the activation energy from 23 meV (associated with thermal broadening of the leads and coherent tunneling) to 257 meV (which is associated with incoherent tunneling). This finding has profound implications in charge transport studies because it shows that polarizable molecules can readily stabilize charge enough to induce thermal activation without the need for formal redox reactions^3,9,28^ or redox-active groups.^9,21,29^

In the coherent tunneling regime, the magnitude of the measured tunneling rate (or current) across molecular junctions depends on the tunneling barrier width (*d*), height (δ*E*_ME_), and molecule—electrode coupling strength (γ) (which can readily be described by single-level tunneling models).^20,30^ For junctions with linear potential drops, often the general tunneling equation is used to determine the tunneling decay coefficient β:
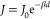
1where *J* is the measured current density across the junction and *J*_0_ is the pre-exponential factor. The charge transport rate (or *J*) in this regime is usually temperature-independent, but weak temperature dependencies have been observed (*E*_a_, the activation energy, of a few tens of meV) which can be assigned to thermal broadening of *E*_F_.^31^ For redox-active junctions, *J* depends on temperature *T* (in K) following the Arrhenius equation
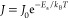
2where *k*_B_ is Boltzmann constant (*k*_B_ = 8.62 × 10^–5^ eV K^–1^).^21^ Incoherent tunneling is expected to be very temperature sensitive because it involves formal oxidation, which requires molecular reorganization to accommodate the charge.^18,21,25,31,32^ Under wet electrochemical conditions, such reorganization processes also involve outer-sphere processes and values of *E*_a_ can be high (1 eV or higher),^33^ but in molecular junctions image charges in the electrodes can compensate for the charge on the molecule and the value of *E*_a_ can be much lower (tens to hundreds of meV).^21,31,32^ Hence, it can be very challenging to discriminate between these charge transport regimes.

A change in the charge transport mechanism from coherent to incoherent tunneling with appreciable values of *E*_a_ (tens to hundreds of meV) can take place by introducing redox-active functional groups, such as ferrocenyl (Fc),^21,31^ bipyridyl,^34^ methylviologen derivatives,^35^ azulene,^36^ metal complexes like [RuIIL_2_](PF_6_)_2_ (L = 2,6-bis(phenylazo)pyridine),^37^ and bipyridine–metal complexes.^38^ In principle, increasing *d* should also lead to a transition from coherent to incoherent tunneling, which has been observed.^10,24−27,39^ Indeed, junctions with conjugated redox-active molecular wires, such as oligophenylene ethynylenes (OPE),^26^ oligophenylenethiopheneimine (OPTI),^25^ and oligophenyleneimine (OPI),^24^ show a very sharp transition from coherent to incoherent tunneling at around *d* = 4 nm.

Although in these examples the mechanism of charge transport is well-defined, very low β values have been reported for nonconjugated molecular wires (0.53 Å^–1^ for oligoglycene and 0.20 Å^–1^ for oligoglycol monolayers).^23,40,41^ We have reported a gradual change from coherent to incoherent charge transport characterized by a *gradual* increase in *E*_a_ from 9 to 58 meV for oligoglycene and oligoglycol SAMs.^23^ The behavior of these molecules falls somewhere in between that of short conjugated molecules with β = 0.2–0.5 Å^–1^ where coherent tunneling dominates and long conjugated wires with *E*_a_ = 0.14–0.65 eV where incoherent tunneling dominates.^24−27,42,43^ Interestingly, *E*_a_ seems to follow a linear relationship with the increase of dielectric constant, ε_r_, which, in turn, increases with increasing *d* for these two systems.^23^ Although the origin of the increase in ε_r_ is unclear, these observations imply that *polarizability*, besides redox activity, of the molecules is an important factor to consider in explaining temperature effects.

Though polarizable α groups can change the measured tunneling rates dramatically,^20,44,45^ it does not need to affect the measured relative dielectric constant ε_r_ because of depolarization effects.^46^ For instance, we have shown across junctions derived from SAMs of S(CH_2_)_*n*_X (X = H, F, Cl, Br, or I) that β decreased from 0.75 (for X = F) to 0.25 Å^–1^ (X = I) along with a factor of 4 increase in ε_r_.^20^ In contrast, junctions with a conjugated backbone SPhX and SPh_2_X (Ph = phenyl ring) exhibit indistinguishable current densities^46,47^ and dielectric behavior^46^ due to collective electrostatic effects.^44,48,49^ Therefore, the relation between ε_r_ and α of the monolayer (since α is the microscopic origin of ε_r_) does not go hand in hand, per se go hand-in-hand, leading to polarizable monolayers yet with low ε_r_.^46^ Therefore, the role of α of the system on the charge transport properties is challenging to isolate.

In this work, we explored a series of aliphatic monolayers with phenyl (Ph) termini, which allows us to study both the effect of the type and number, *n*, of halogen substituents (HS(CH_2_)_10_OPhX_*n*_, X = F, Cl, Br, or I; *n* = 0, 1, 2, 3, or 5). By increasing α going from F to I and by increasing *n*, we show the value of *E*_a_ can be increased from 22 ± 5 meV, a value that can be assigned to both thermal broadening of the leads and coherent tunneling, to 257 ± 22 meV, a value associated with a redox process and incoherent tunneling. The major conclusion of this work is that polarization plays a major role in charge transport, blurring the distinction between coherent and incoherent charge transport regimes. Our findings shed a new perspective on our understanding of the temperature-dependent behavior and associated charge transport mechanisms of molecular junctions, highlighting the importance of polarization of molecules in the mechanism of charge transport. Although polarization is an integral part of the Marcus theory,^50^ our results show that this also holds in solid-state junctions lacking solvent molecules.

## Results and Discussion

### Design of the Molecular Junctions

**Figure 1:**
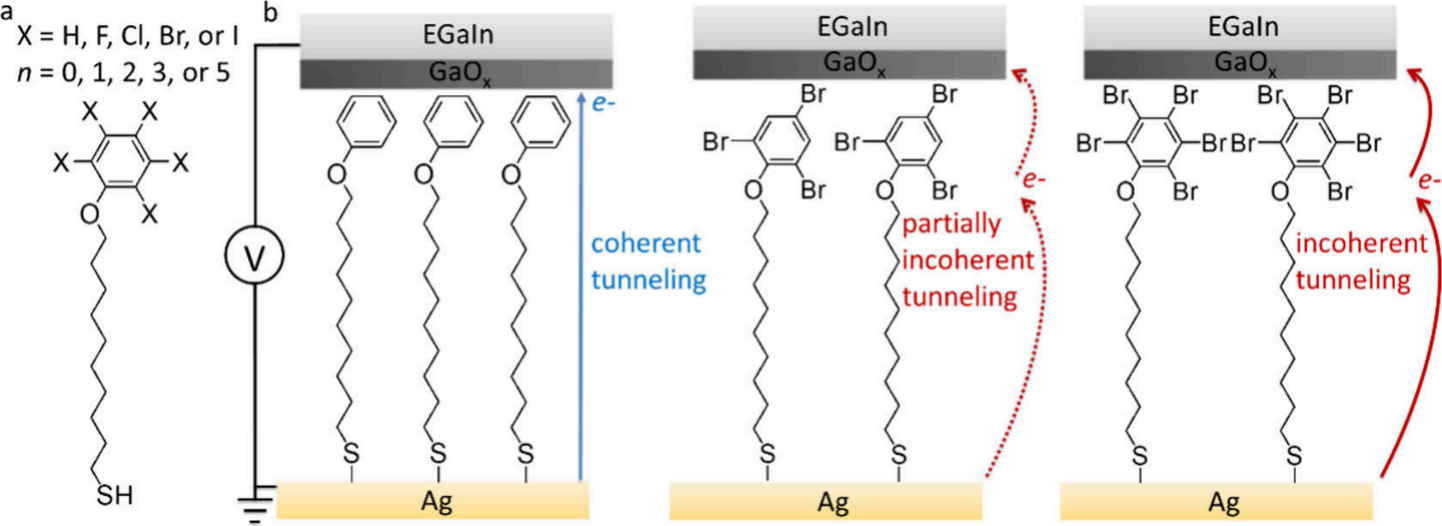
(a) Molecule structure with numbers *n* and halides X used in this work. (b) Schematic illustrations of the Ag–S(CH_2_)_10_OPhBr_*n*_//GaO_*x*_/EGaIn junctions (*n* = 0, 3, and 5) where the bottom electrode Ag (which was grounded) was obtained by a template-stripping method (Supporting Information Section S2).^21^ “//” indicates a noncovalent contact, and GaO_*x*_/EGaIn indicates the top electrode where EGaIn stands for eutectic alloy of gallium and indium with its native oxide layer of GaO_*x*_. The arrows indicate coherent tunneling (solid blue), partially coherent tunneling (dashed red), and incoherent tunneling (solid red) depending on *n*.

Figure 1a shows the structures of all molecules used in this paper, and the synthetic details and characterization of the molecules are given in the Supporting Information (Section S1). All molecules have a thiol anchoring group, an alkyl chain backbone, and a Ph terminal group with a varying number (*n*) of halide substituents (X) abbreviated as HS(CH_2_)_10_OPhX_*n*_. The asymmetrical location of the PhX_*n*_ functional group at the terminal of SAM helps introduce a molecular orbital asymmetrically into the junction and improves the rectification.^21,29,34,35^ To systematically understand how α affects the charge transport properties of the junctions, for *n* = 1 and 3, we investigated the series with X = H, F, Cl, Br, or I, where α increases going from F to I. We studied a second series for X = F and Br to establish how *n* = 0, 1, 2, 3, and 5 affect the junction properties of the junctions in detail. Figure 1b shows the schematic illustration of Ag–S(CH_2_)_10_OPhBr_*n*_//GaO_*x*_/EGaIn with a cone-shaped EGaIn tip that was introduced using previously reported methods (Supporting Information Section S2).^20,21^ In all of our experiments, the bottom Ag electrode was grounded, and the top EGaIn electrode was biased.

### Monolayer Characterization

We characterized the surface properties of two series of Ag–S(CH_2_)_10_OPhX_3_ and Ag–S(CH_2_)_10_OPhBr_*n*_ SAMs using angle-resolved X-ray photoelectron spectroscopy (AR-XPS), ultraviolet photoelectron spectroscopy (UPS), and near-edge X-ray absorption fine structure spectroscopy (NEXAFS) following the previous methods^21,51^ to analyze the SAM packing quality and to establish the trends in energy level alignment of the SAMs on Ag. All results are plotted in Section S3, and Table S1 summarizes the monolayer properties.

**Figure 2:**
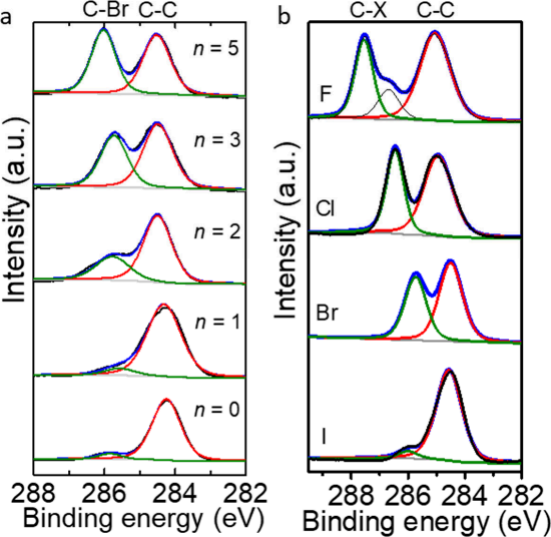
(a) C 1s spectra of Ag–S(CH_2_)_10_OPhBr_*n*_ SAMs and (b) Ag–S(CH_2_)_10_OPhX_3_ SAMs. The two peaks represent two different C chemical environments: solid red lines show C 1s of C–X while the solid green lines show C 1s of C–C. The black lines show the experimental data, and the solid blue lines show the corresponding fit.

First, we discuss the XPS results of SAMs of Ag–S(CH_2_)_10_OPhBr_*n*._Figure 2a shows the C 1s spectra for *n* = 0, 1, 2, 3, and 5, which are dominated by a peak at 284.4 eV with contributions from C–C and C=C^20,46^ (solid red line), and a small peak at 286.2 eV for *n* = 0 which is attributed to C–O^23^ (solid green line). This small peak increases with increasing number of Br substituents, indicating overlap with the C–Br signal (see also Figure S5).^20,46^ The peak at 286.2 eV dominates over the peak at 284.4 eV for *n* = 5, which indicates that all Br substituents are located at the top of the SAM. Figure 2b shows the C 1s spectra for Ag–S(CH_2_)_10_OPhX_3_ SAMs and a clear shift in the C–X signal from 286.1 eV for X = I to 287.7 eV for X = F due to increasing electronegativity of X, which agrees with previous reports.^20,46^

The S 2p spectra (Figure S6) are dominated by a single doublet of S 2p_1/2_ and S 2p_3/2_ (with spin–orbit splitting (SOS) of 1.18 eV) with the S 2p_1/2_ peak centered at ∼161.8 eV.^20,46^ These characteristics are associated with chemisorbed S commonly reported for *n*-alkanetiolate SAMs and indicate that the SAMs did not suffer from obvious physisorption or disorder. Figure S7 shows the Br 3d spectra dominated by a single doublet (with SOS of 1.05 eV) as expected.^20,46^ The signal intensity does not change significantly with decreasing angle from 90° to 40°, which confirms that the Br atoms are at the terminal position of the SAM. To confirm the structure of other halide SAMs, we also recorded the angle-resolved XPS data of the SAMs as a function of X for *n* = 3 (Figures S9–S11). The S 2p signal indicates the lack of physisorbed materials, and both the C 1s and F 1s, Cl 2p and I 3d spectra confirm the presence of the halide. From all these XPS data, we conclude that the monolayers are well-ordered, and they were stable under the experimental conditions despite the large number of halides for *n* = 5.

We also derived the relative surface coverage (Γ_SAM_) to the SAM with *n* = 0 from the ratio of S and Ag peak intensity and the SAM thickness (*d*_SAM_) from the AR-XPS data.^21^ All of the Ag–S(CH_2_)_10_OPhX_*n*_ SAMs show similar *d*_SAM_ and Γ_SAM_ (Tables S1 and S2) within error. From the angle-resolved NEXAFS data, we found that the tilt angle of the phenyl group for all SAMs^51^ is in the range of 25°–33° (well within the ±5° error). From these data, we conclude that all SAMs have a similar supramolecular organization (thus eliminating potential differences in SAM organization as a possibility to explain the differences in charge transport characteristics discussed below).

### Monolayer Electronic Structure

**Figure 3:**
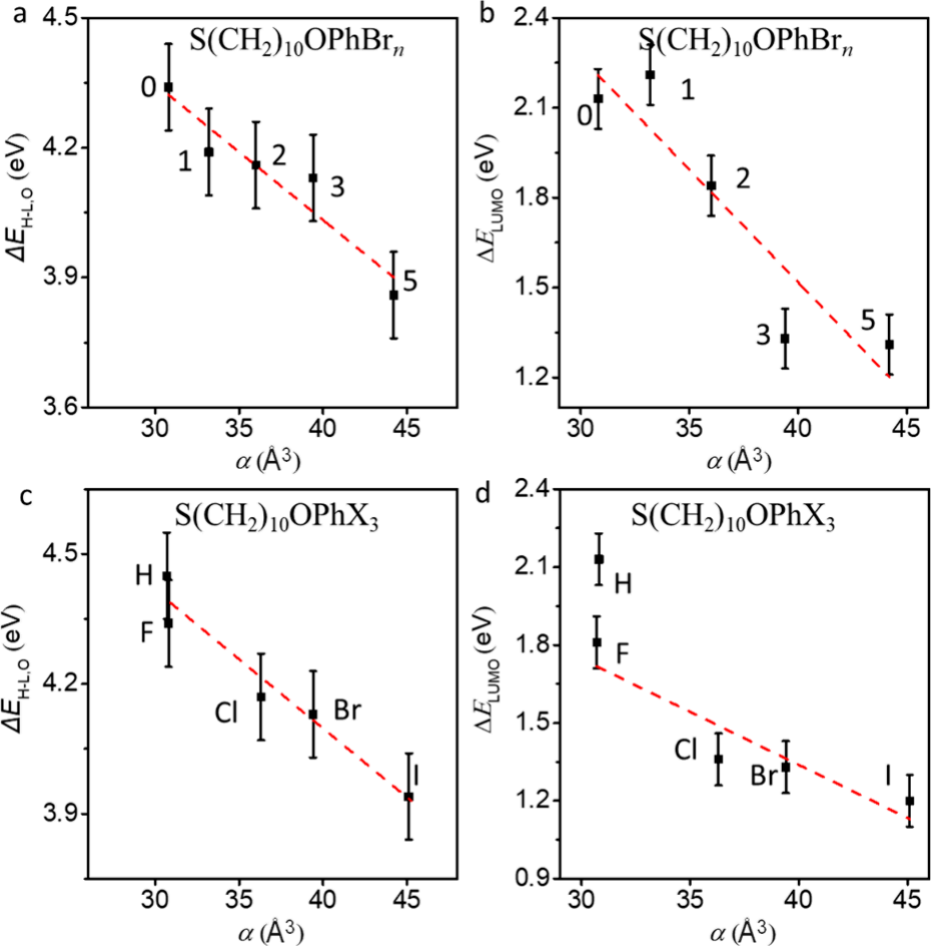
Δ*E*_H–L,O_ plotted against α for S(CH_2_)_10_OPhBr_*n*_ (a) and S(CH_2_)_10_OPhX_3_ (c) SAMs on Ag; Δ*E*_LUMO_ plotted as a function of α for S(CH_2_)_10_OPhBr_*n*_ (b) and S(CH_2_)_10_OPhX_3_ (d) SAMs on Ag. The numbers and atoms on the panels indicate the *n* or X of the SAMs. The red dashed lines are visual guides.

To establish the energy level alignment of all the SAMs (see energy level diagrams in Figure 6), we characterized the energy offset between HOMO energy (*E*_HOMO_) and Fermi level *E*_F_ (Δ**E**_HOMO_) using UPS (Figure S12) and determined the optical HOMO–LUMO gap (Δ*E*_H–L,O_) using UV–vis spectroscopy^52^ (Figure S15, Table S3 lists all results), which can be used to estimate the energy of the LUMO (*E*_LUMO_).^21,51^ Using density functional theory (DFT; the B3LYP method, and the CEP-31G** basis), we calculated the value of α of the molecules in the gas phase. Figure 3 shows the evolution of Δ*E*_H–L,O_ and Δ*E*_LUMO_ as a function of α of S(CH_2_)_10_OPhX_*n*_, and the rest of the data are given in Figures S15 and S16. Both Δ*E*_H–L,O_ and Δ*E*_LUMO_ (the energy gap between LUMO and *E*_F_) decrease with increasing *n* of halide substituents or with X going from F to I. These trends are very well-known to occur^5,53,54^ and can be explained by the fact that a polarizable material can readily stabilize electrons.^54−56^ In contrast, Δ*E*_HOMO_ is independent of *n* and X (Figure S16) likely due to Fermi-level pinning.^30,57−59^

### Electrical Characterization of Molecular Junctions

**Figure 4:**
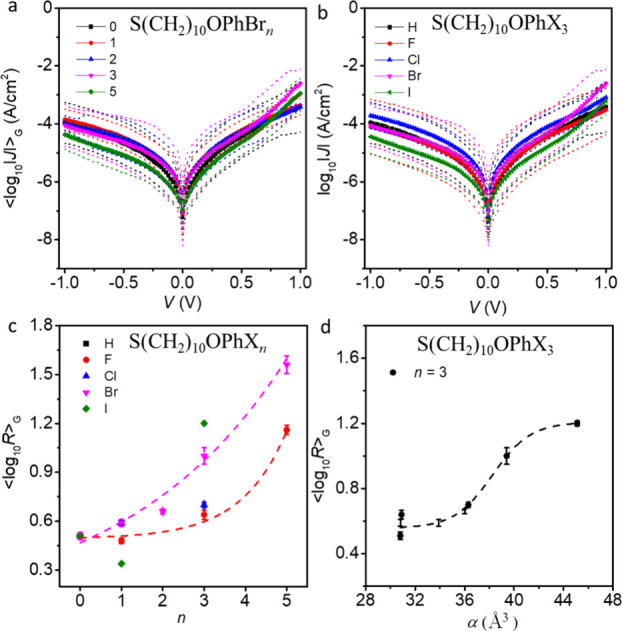
⟨log*|J|*⟩_G_ vs *V* curves of (a) Ag–S(CH_2_)_10_OPhBr_*n*_//GaO_*x*_/EGaIn junctions and (b) of Ag–S(CH_2_)_10_OPhX_3_//GaO_*x*_/EGaIn junctions. (c) ⟨log *R*⟩_G_ vs *n* for all Ag–S(CH_2_)_10_OPhX_*n*_//GaO_*x*_/EGaIn junctions at ±1.0 V. (d) Plot of ⟨log *R*⟩_G_ vs α for Ag–S(CH_2_)_10_OPhX_3_ junctions. The dashed lines are guides to the eyes.

The junctions were characterized with current density—voltage (*J*(*V*)) measurements, impedance spectroscopy (to determine the capacitance of the junctions and ε_r_), and temperature-dependent *J*(*V*) measurements following previously reported methods^20,21^ (see Section S5 for details). We recorded 373–456 *J*(*V*) curves for each type of junction from which we determine the Gaussian log-average value of *J*⟨log*|J|*⟩_G_, and associated log-standard deviation, for each measured value of *J.*Figure 4a,b shows the ⟨log*|J|*⟩_G_ vs *V* curves recorded from the representative SAMs of Ag–S(CH_2_)_10_OPhBr_*n*_//GaO_*x*_/EGaIn and Ag–S(CH_2_)_10_OPhX_3_//GaO_*x*_/EGaIn junctions for *V* = ±1.0 V, respectively. The dashed lines are the 95% confidence levels, showing that all junction data fall within this error window. Only junctions with *n* ≥ 3 and X = Br or I show appreciable rectification ratios of ≥10 at ±1 V. The mechanism behind this rectification will be explained in more detail below.

Figure 4c shows the ⟨log *R*⟩_G_ at ±1.0 V for all Ag–S(CH_2_)_10_OPhX_*n*_//GaO_*x*_/EGaIn junctions. We observed that for the same value of *n* (i.e., *n* = 3 and 5), ⟨log *R*⟩_G_ increases from X = H to X = I. In addition, for the same X (i.e., X = F and Br), ⟨log *R*⟩_G_ increases with *n* (see dashed pink and red lines as visual guides). Figure 3 shows the relation between electronic structure and α from which we conclude that the rectification performance directly relates to changes in α and associated changes in Δ**E**_LUMO_. For example, Figure 4d shows the plot of ⟨log *R*⟩_G_ against α for Ag–S(CH_2_)_10_OPhX_3_//GaO_*x*_/EGaIn junctions with varying X from H and F to I to highlight how polarization effects induce electronic function (see Figure S23 for additional plots).

### Impedance Spectroscopy

Normally, one would expect that ε_r_ increases with α,^20,48,60^ but due to the depolarization effects ε_r_ may remain unchanged despite changes in α.^46,60^ We used impedance spectroscopy at 0 V (using a sinusoidal perturbation of 30 mV in the frequency range of 1 to 1 × 10^6^ Hz) to separate the contribution of each element of the junctions, i.e., the resistance of the SAM (*R*_SAM_ in Ω·cm^2^), the resistance of the contact (*R*_C_ in mΩ·cm^2^), and the capacitance of the SAM (*C*_SAM_ in μF/cm^2^), following previously reported methods.^20,44^Figures S24–26 show the Nyquist, Bode, and phase plots, and Tables S7–S9 summarize the fitting results. These results show that the number and type of halide substituents did not affect the value of *R*_C_, from which we conclude that the halide substituent did not affect the SAM//top contact interface. The observation that *R*_SAM_ is hardly affected around 0 V confirms that all junctions have a similar monolayer thickness and the same mechanism of charge transport at around 0 V, that is, off-resonant tunneling; below we show that the mechanism of charge transport depends on the applied voltage. For all SAMs, the values of *C*_SAM_ (∼1.2 μF/cm^2^) and ε_r_ (∼2.4) are similar, from which we conclude that one phenyl ring is sufficient to induce collective electrostatic effects that result in a similar measured capacitance despite differences in α.^46,47^

### Temperature-Dependent *J*(*V*,*T*) Characterization

**Figure 5:**
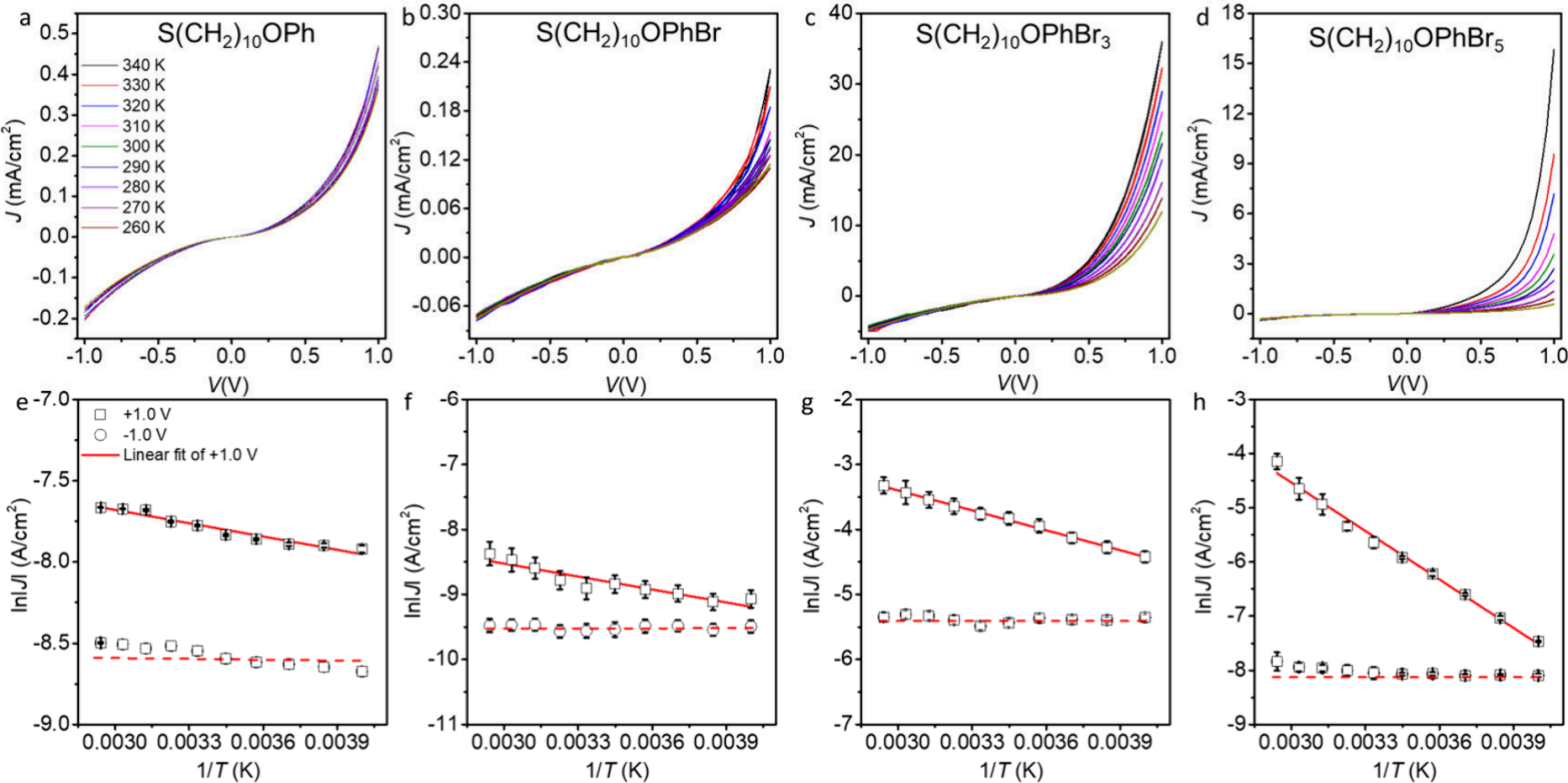
Representative *J*(*V*) curves of Ag–S(CH_2_)_10_OPhBr_*n*_//GaO_*x*_/EGaIn junctions in the range of *T* of 250–340 K (a–d). Corresponding Arrhenius plots at +1.0 and −1.0 V (e–h). Red solid lines are fits to eq 2, and the red dashed lines are guides to the eyes. The error bars represent the standard deviation from 3 different measurements at each temperature.

To determine the mechanism of charge transport in this series of molecular junctions, we carried out the *J*(*V*,*T*) measurements over a *T* range of 250–340 K. Figure 5 shows *J*(*V*,*T*) for the representative junctions of Ag–S(CH_2_)_10_OPhBr_*n*_//GaO_*x*_/EGaIn for *n* = 0, 1, 3, and 5 along with the Arrhenius plots at −1.0 and +1.0 V (the rest of the data are shown in Figures S27–S30). The Arrhenius plots show that at a negative bias of −1.0 V, |*J*| is almost constant, while at +1.0 V, |*J*| decreases with *T*. The red solid and dashed lines are fits of the data to the Arrhenius equation (eq 2), indicating that coherent tunneling dominates at −1.0 V and incoherent tunneling dominates at +1.0 V. In general, coherent tunneling is more efficient than incoherent tunneling (hopping) at short distances; over long distances incoherent tunneling becomes the dominant mechanism of charge transport as has been shown by varying the length of the molecule.^24,25,27^ Besides length, the energy level alignment of the system also plays a crucial role. Since in our junctions we did not change *d*_SAM_, our results show that thermally assisted incoherent tunneling is more efficient in charge transport than temperature-independent coherent tunneling, resulting in higher current in only one bias direction and, thus, in rectification, in agreement with prior works.^21,29,34^ Thus, junctions with different charge transport mechanisms at positive and negative bias are an interesting way to yield good rectifiers (with rectification ratios of 3–5 orders of magnitude).^18,21,35,61,62^ In principle, junctions with coherent tunneling in both bias directions can lead to appreciable rectification ratios of 2–3 orders of magnitude,^63^ but in practice lower rectification ratios (<100) are found.^20,34,46^

**Figure 6:**
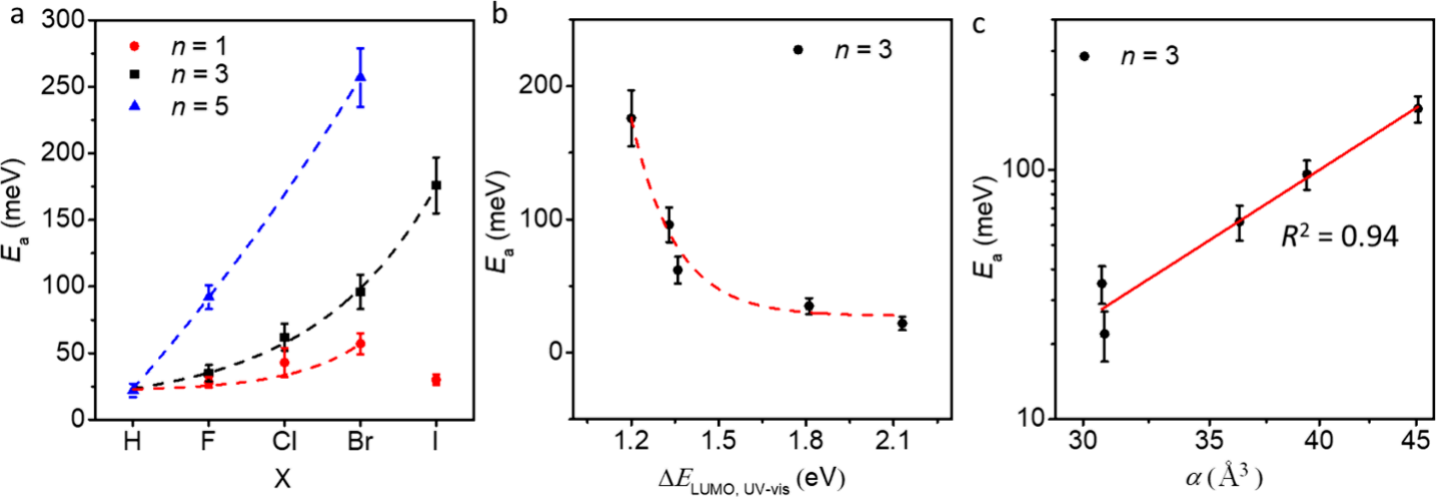
(a) *E*_a_ of Ag–S(CH_2_)_10_OPhX_*n*_//GaO_*x*_/EGaIn vs *T* = 250–340 K. Plots of *E*_a_ vs Δ*E*_LUMO_ (b) and *E*_a_ vs α (c) for junctions of Ag–S(CH_2_)_10_OPhX_3_//GaO_*x*_/EGaIn. The error bars are the standard deviation from three measurements. The dashed lines are guides to eyes. The linear lines are linear fits of the plots.

Figure 6a shows a summary of *E*_a_ vs X for *n* = 1, 3, and 5. These plots show that *E*_a_ increases with X and *n*, and thus also with α. For example, *E*_a_ values range from 23 ± 2 meV for X = H to 176 ± 11 meV for X = I and *n* = 3. For junctions with *n* = 5 and X = F, *E*_a_ is 92 ± 9 meV but increases to 257 ± 22 meV for X = Br and *n* = 5. Such large values of *E*_a_ are normally found for wet electrochemical redox processes or molecular junctions with redox active molecules (see the Introduction), yet our junctions are not redox-active (see electrochemical characterization of the precursor Br(CH_2_)_10_OPhBr_5_ in Figure S31). To demonstrate that *E*_a_ correlates with α (and associated changes in Δ**E**_LUMO_), Figure 6b,c shows *E*_a_ as a function of Δ**E**_LUMO_ and α for Ag–S(CH_2_)_10_OPhX_3_//GaO_*x*_/EGaIn junctions. Interestingly, *E*_a_ is much more affected at positive bias than at negative bias where its value is small and seems to be independent of X and *n* (Figure 6a). These observations imply that the LUMO plays a pivotal role in the mechanism of charge transport, leading to the gradual change from coherent to incoherent tunneling. At opposite bias, both the HOMO and LUMO do not fall in the bias window over the range of applied *V*, and off-resonant tunneling dominates (see the next section for details).

These observations confirm that the LUMO plays an important role in the mechanism of charge transport, where the increase in α enables thermally activated charge transport in part by decreasing the tunneling barrier height of Δ**E**_LUMO_. We would like to emphasize that the value of *E*_a_ is not only defined by the barrier height as *E*_a_, but that in our series of experiments the reorganization energy also changes. As discussed before, the charge transport mechanism is often switched by adding redox centers^21,29,31,35−38^ or increasing the molecular length of conjugated backbones,^24−27^ but these two mechanisms normally cause an abrupt transition from coherent to incoherent tunneling. Our results show that increasing the value of α of SAMs with nonconjugated backbones can result in gradual transition of the charge transport mechanism from coherent to incoherent tunneling with *E*_a_ of several tens of meV to a few hundreds of meV.^23^ Therefore, we are able to tune the charge transport mechanism without changing the molecular length and/or the introduction of formal redox centers. In the incoherent tunneling regime, the charge carrier fully relaxes on the molecule, leading to a formal change in redox state and loss of coherence^21,25,29,64^ while the opposite holds true in the coherent tunneling regime. Our results imply that by increasing the polarizability of the molecules, the interaction with the charge carrier is increased, leading to a thermally activated component. Our results infer that it is possible that a new intermediate mechanism takes place with partial loss of coherence with the measured *E*_a_ relating to polarization^23^ and representing an effective reorganization energy required for charge carriers to interact with the functional head groups.

### Charge Transport Mechanism

**Figure 7:**
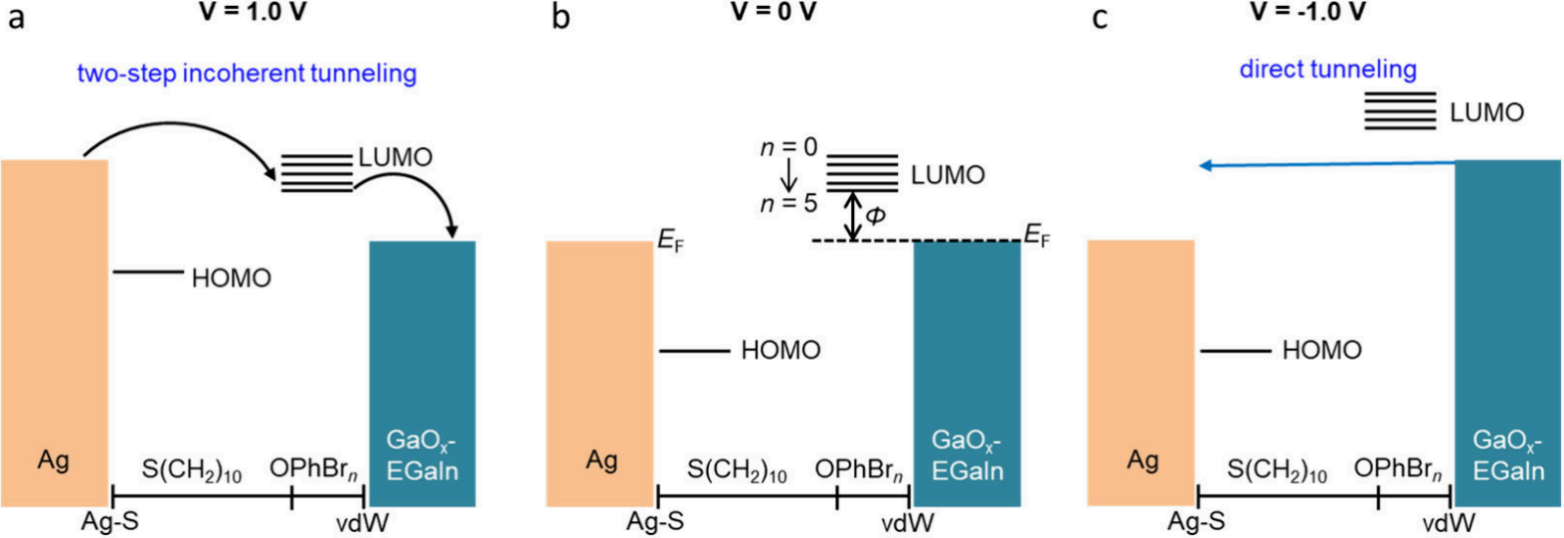
Energy level diagrams showing the positions of the molecular orbitals relative to the electrodes under positive bias of 1.0 V (a), equilibrium (b), and negative bias of −1.0 V (c) for Ag–S(CH_2_)_10_OPhBr_*n*_//GaO_*x*_/EGaIn junctions. The Δ**E**_LUMO_ decreases with *n* as indicated in panel b. Note, in our experiments we grounded the Ag electrode and applied the bias to the top electrode.

As mentioned in the Introduction, junctions with asymmetrical positioned redox-active moieties such as fulleropyrrolidines,^65^ diarylethene–bisthienylbenzene,^66^ naphtalenediimide,^67,68^ metallocene,^69^ and S(CH_2_)_11_T (with T = Fc,^21,29,70^ bipyridyl,^34^ or bipydine–MCl_2_ (M = Co, Mn, Fe, or Ni)^38^) are good molecular diodes, but junctions with asymmetrical molecules that are polar or conjugated but non-redox-active (e.g., T = halogens,^20^ biphenyl, phenylpyridyl, and pyrazinyl^34^) tend to operate in the coherent coherent tunneling in both bias directions and show very low or negligible rectification. Figure 7 shows the energy level diagrams for the Ag–S(CH_2_)_10_OPhBr_*n*_//GaO_*x*_/EGaIn junctions based on the data in Table S3 and how the energy of the LUMO shifts toward the *E*_F_ of the top electrode with increasing polarizability or number of substituents. The HOMO is centered around the sulfur atom,^30,57,58^ and Δ*E*_HOMO_ values (Figure S16) are similar for all SAMs due to the Fermi-level pinning caused by the strong pinning effect of the S–Ag bond.^30,57−59^ For this reason, HOMO will follow the potential of the bottom electrodes in our experiments. In contrast, the LUMO is centered at the top of the monolayer and in close contract with the top electrode. Since most of the potential drops along the alkyl chain, the LUMO will follow the changes of the potential of the top electrode.^21,29,71^ As indicated in Figure 7, we grounded the bottom electrode and applied the bias to the top electrode where at 0 V the LUMO is above the Fermi level. When a positive bias of 1.0 V is applied, the LUMO follows the change in the energy of the top electrode and is lowered so that it can fall in the conduction window. The contribution of the LUMO to charge transport is that the LUMO could serve as hopping sites for charge carriers,^21,25,29,64^ leading to a change in the mechanism of charge transport from coherent to incoherent tunneling. Although we do not know how strongly the LUMO couples with the top electrode, since this contact is a van der Waals contact, we presumed a voltage drop of 0.3 eV at the SAM//top-electrode interface following prior works.^21,29^ At the opposite bias, the LUMO cannot fall in the conduction window (at least in the applied bias range; at much larger negative applied bias, the LUMO can in principle enter the bias window as well). Therefore, the LUMO is only accessible for charge transport at positive bias, which results in the rectification behavior similar to other LUMO-mediated molecular diodes including bipyridyl^34^ or bipyridine–metal complexes^38^ which rectify at positive bias with *R* of >80. Our results showcase that polarization plays a crucial role (in line with the Marcus theory^50^) in the mechanism of charge transport, leading to appreciable temperature effects and, in turn, to rectification in solid state tunnel junctions.

## Conclusions

By introducing halides with different numbers and at different positions to a phenyl moiety in large-area junctions, we tuned *R* and *E*_a_ without changing the molecular backbone, length, and ε_r_. Remarkably, we were able to access a charge transport regime in between the coherent (essentially no thermal activation) and incoherent tunneling regimes (large activation energies) without changing the molecular length (unlike in the case of peptides,^23^ proteins,^11,72^ or conjugated backbones^24−27^) or redox centers. By only changing the number and type of halide substituent, we changed the activation energy gradually by more than 1 order of magnitude from 22 to 257 meV. Therefore, we conclude that the mechanism of charge transport for polarizable molecules may be in between coherent and incoherent tunneling regimes.

We have shown before that halides can affect the observed tunneling rates by 3–4 orders of magnitude across monolayers of the form S(CH_2_)_*n*-1_CH_2_X, but in those studies the molecular frontier orbitals were far away from the conduction window and charge transport was activationless.^20^ In this work, we establish that a molecular orbital must be available to transit the mechanism from the coherent to the incoherent tunneling regime. In the present study, the LUMO was energetically available but only in one bias polarity, leading to substantial rectification. Our results show that by introducing polarizable moieties, appreciable rectification ratios of 40 can be achieved. Although these are not the largest rectification ratios, our results suggest that the electronic function of molecular devices can be improved by considering polarization effects.

Although the dielectric constant is the macroscopic manifestation of polarization, due to depolarization effects^46^ the measured value of ε_r_ remains unaltered by (large) changes in α. We showed that despite the introduction of 5 halide atoms, ε_r_ did not change. Yet, the mechanism of charge transport changed from coherent to incoherent tunneling, proving that monolayers can provide a highly polarizable medium without changing ε_r_. Therefore, in solid state junctions it is important to consider α and not only ε_r_, as also has been suggested before theoretically^12,48^ to explain high tunneling rates,^20^ long-range tunneling,^10,11^ or CISS effects.^12−14^

On a final note, molecular tunneling junctions are usually modeled with tunneling models (Landauer theory^20^) or combined with Marcus theory for redox-active junctions^18,21^ in case thermally activated transport is significant; this work shows that a transition region exists for which a theoretical framework is not available. For all of these reasons, we believe that our results are important for future investigations of tunneling rates and help the community to establish how temperature affects the charge transport.

## Supporting Information Available

Details of the synthesis and NMR characterization of the molecules; preparation of the Ag substrates and SAMs; XPS, UPS, and NEXAFS characterizations of the SAMs on Ag; optical gaps and DFT calculations; electrical *J*(*V*), impedance, and temperature-dependent characterization of the junctions (PDF)
