# Supplementary material for: Gradual Change between Coherent and Incoherent Tunneling Regimes Induced by Polarizable Halide Substituents in Molecular Tunnel Junctions
Source: J Am Chem Soc. 2024 Aug 8;146(33):23356–64. doi: 10.1021/jacs.4c06295 (PMC11345807; doi:10.1021/jacs.4c06295)
Supplement: Supplementary file 1 — ja4c06295_si_001.pdf [file ja4c06295_si_001.pdf]

Supporting Information for

Gradual Change between Coherent and Incoherent Tunnelling Regimes Induced by Polarizable Halide Substituents in Molecular Tunnel Junctions

Xiaoping Chen[1,2], Ira Volkova[2], Yulong Wang[2],Ziyu Zhang[2], and Christian A. Nijhuis[2,3,4]*

[1]College of Chemistry, Chemical Engineering and Environment, Fujian Provincial Key Laboratory of Modern Analytical Science and Separation Technology, Minnan Normal University, Zhangzhou, 363000, China.

[2]Department of Chemistry, National University of Singapore, 3 Science Drive 3, Singapore 117543, Singapore

[3]Centre for Advanced 2D Materials and Graphene Research Centre, National University of Singapore, 6 Science Drive 2, Singapore 117546, Singapore

[4]Hybrid Materials for Opto-Electronics Group, Department of Molecules and Materials, MESA+ Institute for Nanotechnology and Molecules Centre, Faculty of Science and Technology, University of Twente, 7500 AE Enschede, The Netherlands

*Author to whom correspondence: c.a.nijhuis@utwente.nl

Section S1: Materials and Synthesis of Molecules

Materials. All the regents and chemicals were purchased from Sigma-Aldrich or Tokyo Chemical Industry CO., Ltd except specifically mention. Chemicals and solvents were directly used without further purification or treatment. Deionized water was collected using Elga Purelab option-Q system. Silica gel (60Å/40-63 µm) was obtained from Sigma-Aldrich. ^{1}H and ^{13}C NMR spectra were recorded from Bruker Avance 300 MHz spectrometer or Bruker Avance 400 MHz spectrometer with $CDCl_3$ as solvent. Electron ionization (EI) mass spectra were collected from Finnigan LCQ mass spectrometer. High resolution electron ionization mass spectra (EI-HR-MS) were collected from Bruker microTOF-QII mass spectrometer. CHI 760E electrochemical workstation (Shanghai Chenhua instrument Co., Ltd., China.) equipped with a three-electrode system was used to conduct electrochemistry measurement.

Synthesis Scheme. The synthesis routes are shown in Figure S1. The synthesis methods for $Br_nPhO(CH_2)_{10}SH$ with n=0 to 3 were the same, the pentabromoderive was differently prepared as shown. Here, we gave details the synthesis of $PhO(CH_2)_{10}SH$ and $Br_5PhO(CH_2)_{10}SH$ as examples. The associated NMR spectra of all the thiolated molecules are shown in Figures S2-4.

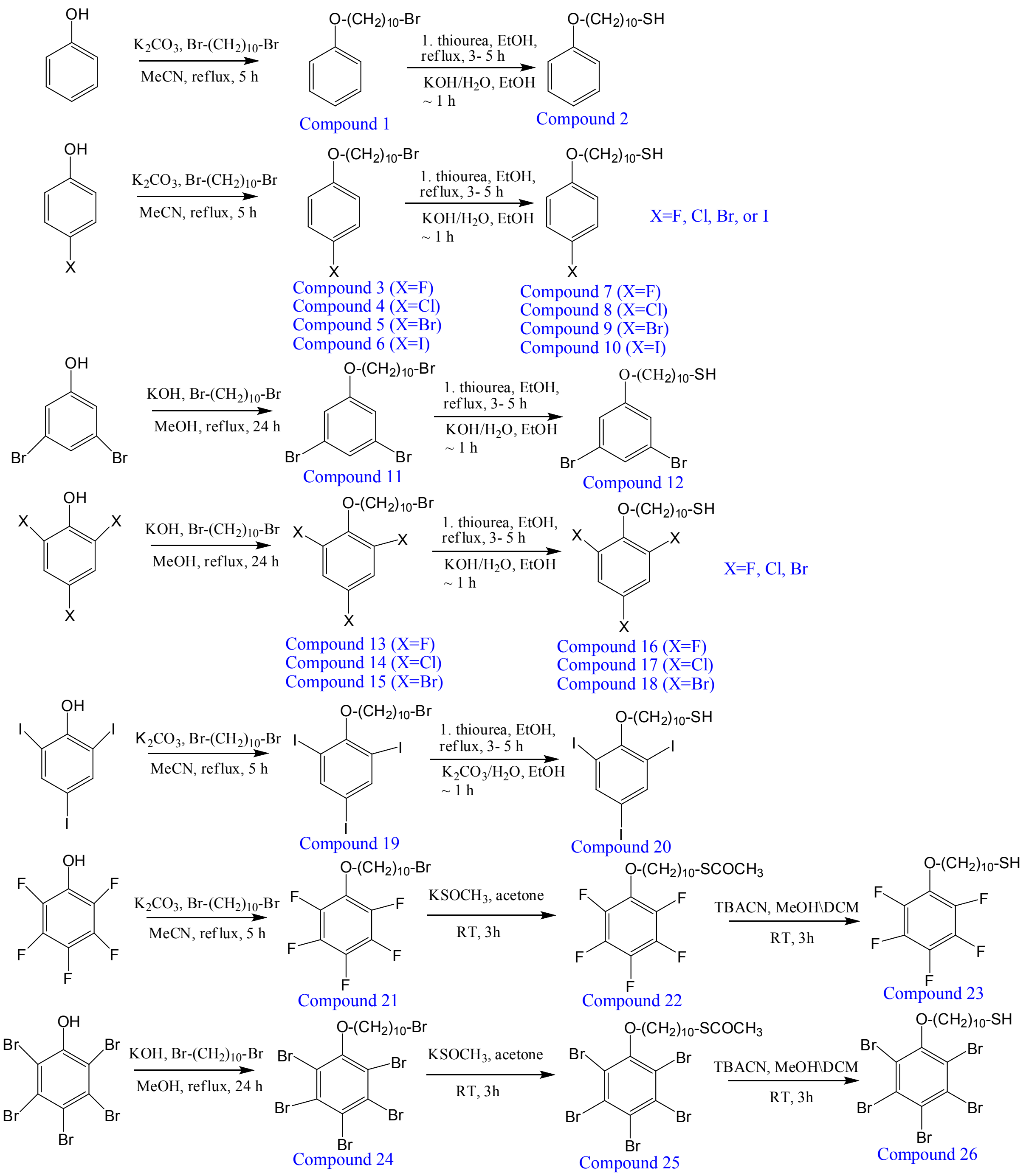

Figure S1. The synthetic scheme of the $X_nPhO(CH_2)_{10}SH$ derivatives used in this study.

Synthesis of $PhO(CH_2)_{10}Br$ (Compound 1). The synthesis of $PhO(CH_2)_{10}Br$ followed a slightly altered reported procedure[1]. We added 3.00 g (32 mmol) phenol, 19.20 g (64 mmol), $Br(CH_2)_{10}Br$, and 3.59 g (64 mmol) KOH in ~100 methanol in a two-neck round flask under N_2. The mixture was refluxed for 24 h before cooling down to room temperature. The solvent

was removed by rotary evaporator and dissolved in dichloromethane (DCM) and washed with deionized water for three times. The separated organic layers were combined together and dried over anhydrous Na_2SO_4, filtered, and evaporated under reduced vacuum. The crude product was purified on column chromatography with hexane as eluent. The product was collected as the second band with yield of 70 % (7.02 g). ^{1}H-NMR (300 MHz, $CDCl_3$): δ = 1.42-1.53 (m, 12H, -*$(CH_2)_6$*-$(CH_2)_2$Br), 1.90 (m, 4H, -*CH_2*-CH_2O and -*CH_2*-CH_2Br), 3.47 (t, 2H, *J* = 6.9 Hz, -*CH_2*Br), 4.02 (t, 2H, *J* = 6.3 Hz, -*CH_2*-O-), 7.00 (m, 3H, ArH), 7.36 (t, 2H, *J* = 8.0 Hz, ArH) ppm. ^{13}C-NMR (75 MHz, $CDCl_3$): δ = 26.05, 28.15, 28.74, 29.30, 29.35, 29.45, 32.82, 33.83, 67.73, 114.43, 120.37, 129.32, 159.12 ppm. EI-MS: 312.0 $[M]^+$.**The synthesis procedures of Compounds 3, 4, 5, 6, 11, 13, 14, 15, 19, 21, and 24 are similar to Compound 1.**

FPhO$(CH_2)_{10}$Br (Compound 3). ^{1}H-NMR (300 MHz, $CDCl_3$): δ = 1.31-1.43 (m, 12H, -*$(CH_2)_6$*-CH_2CH_2Br), 1.76-1.86 (p, 4H, *J* = 6.6 Hz, *J* = 7.2 Hz, -*CH_2*-CH_2Br and -OCH_2-*CH_2*-), 3.41 (t, 2H, *J* = 6.6 Hz, -*CH_2*Br), 3.91(t, 2H, *J* = 6.6 Hz, -O-*CH_2*-), 6.83 (m, 2H, ArH), 6.96 (m, 2H, ArH). ^{13}C-NMR (75 MHz, $CDCl_3$): δ = 26.14, 28.29, 28.87, 29.42, 29.46, 29.48, 29.56, 32.96, 34.10, 68.78, 115.52, 115.63, 115.68, 115.99, 155.40, 155.42, 155.69, 158.84 ppm. EI-MS: 330.0 $[M]^+$.

ClPhO$(CH_2)_{10}$Br (Compound 4). ^{1}H-NMR (300 MHz, $CDCl_3$): δ = 1.32-1.44 (m, 12H, -*$(CH_2)_6$*-CH_2CH_2Br), 1.77-1.86 (p, 4H, *J* = 6.6 Hz, *J* = 7.2 Hz, -*CH_2*-CH_2Br and -OCH_2-*CH_2*-), 3.41 (t, 2H, *J* = 7.2 Hz, -*CH_2*Br), 3.91 (t, 2H, *J* = 6.6 Hz, -O-*CH_2*-), 6.82 (m, 2H, ArH), 7.22 (m, 2H, ArH). ^{13}C-NMR (75 MHz, $CDCl_3$): δ = 26.10, 28.28, 28.85, 29.31, 29.43, 29.46, 29.54, 32.94, 34.08, 68.42, 115.89, 125.40, 129.36, 157.88 ppm. EI-MS: 347.9 $[M]^+$.

BrPhO$(CH_2)_{10}$Br (Compound 5). ^{1}H-NMR (300 MHz, $CDCl_3$): δ = 1.31-1.43 (m, 12H, -*$(CH_2)_6$*-CH_2CH_2Br), 1.76-1.85 (m, 4H,-*CH_2*-CH_2Br and -OCH_2-*CH_2*-), 3.41 (t, 2H, *J* = 6.9 Hz,

-*CH_2*Br), 3.91(t, 2H, *J* = 6.3 Hz, -O-*CH_2*-), 6.77 (d, 2H, *J* = 9.0 Hz, ArH), 7.36 (d, 2H, *J* = 9.0 Hz, ArH). ^{13}C-NMR (75 MHz, $CDCl_3$): δ = 26.11, 28.30, 28.87, 29.30, 29.44, 29.48, 29.55, 32.96, 34.11, 68.40, 112.72, 116.47, 132.33, 158.41 ppm. EI-MS: exact mass calculated: 392.0 $[M]^+$.

IPhO(CH_2)$_{10}$Br (Compound 6). ^{1}H-NMR (300 MHz, $CDCl_3$): δ = 1.31-1.42 (m, 12H, -*(CH_2)$_6$*-CH_2CH_2Br), 1.76-1.85 (p, 4H, *J* = 6.6 Hz, *J* = 7.2 Hz, -*CH_2*-CH_2Br and -OCH_2-*CH_2*-), 3.41 (t, 2H, *J* = 7.2 Hz, -*CH_2*Br), 3.91(t, 2H, *J* = 6.6 Hz, -O-*CH_2*-), 6.67 (m, 2H, ArH), 7.54 (m, 2H, ArH). ^{13}C-NMR (75 MHz, $CDCl_3$): δ = 26.11, 28.30, 28.88, 29.28, 29.44, 29.48, 29.55, 32.97, 34.13, 68.26, 82.54, 117.11, 138.31, 159.17 ppm. EI-MS: 438.0 $[M]^+$.

Br_2PhO(CH_2)$_{10}$Br (Compound 11). ^{1}H-NMR (300 MHz, $CDCl_3$): δ = 1.31-1.43 (m, 12H, -*(CH_2)$_6$*-CH_2CH_2Br), 1.76-1.86 (m, 4H, *J* = 6.6 Hz, *J* = 6.9 Hz,-*CH_2*-CH_2Br and -OCH_2-*CH_2*-), 3.41 (t, 2H, *J* = 6.9 Hz, -*CH_2*Br), 3.91(t, 2H, *J* = 6.6 Hz, -O-*CH_2*-), 6.98 (d, 2H, ArH), 7.22 (t, 1H, ArH). ^{13}C-NMR (75 MHz, $CDCl_3$): δ = 26.04, 28.30, 28.88, 29.12, 29.37, 29.48, 29.54, 31.74, 32.97, 68.77, 117.11, 123.22, 126.34, 160.52 ppm. EI-MS: 470.0 $[M]^+$.

F_3PhO(CH_2)$_{10}$Br (Compound 13). ^{1}H-NMR (300 MHz, $CDCl_3$): δ = 1.23-1.25 (m, 10H, -*(CH_2)$_5$*-(CH_2)$_2$Br), 1.40-1.45 (m, 2H, -*CH_2*-(CH_2)$_7$Br), 1.69-1.90 (m, 4H, -*CH_2*-(CH_2)$_8$Br and -*CH_2*-CH_2Br), 3.38-3.42 (t, 2H, -*CH_2*Br), 4.02-4.07 (t, 2H, -*CH_2*-(CH_2)$_9$Br), 6.60-6.72 (t, 2H, ArH) ppm. ^{13}C-NMR (75 MHz, $CDCl_3$): δ = 25.55, 28.11, 28.67, 29.17, 29.27, 29.34, 29.81, 32.79, 33.83, 75.07, 100.20, 100.33, 100.43, 100.56, 100.68, 100.79, 100.91, 132.43, 132.50, 132.62, 132.69, 132.82, 132.88, 154.38, 154.48, 154.57, 154.68, 155.10, 155.29, 155.48, 157.68, 157.79, 157.88, 157.98, 158.35, 158.54, 158.73 ppm. EI-MS: 365.9 $[M]^+$.

Br_3PhO(CH_2)$_{10}$Br (Compound 15). ^{1}H-NMR (300 MHz, $CDCl_3$): δ = 1.32-1.52 (m, 12H, -*(CH_2)$_6$*-CH_2CH_2Br), 1.86 (p, 4H, *J* = 6.6 Hz, *J* = 7.2 Hz, -*CH_2*-CH_2Br and -OCH_2-*CH_2*-), 3.41 (t, 2H, *J* = 6.6 Hz, -*CH_2*Br), 3.97(t, 2H, *J* = 6.6 Hz, -O-*CH_2*-), 7.64 (s, 2H, ArH). ^{13}C-NMR

(75 MHz, $CDCl_3$): δ = 25.95, 28.30, 28.88, 29.51, 29.57, 30.10, 32.99, 34.21, 73.85, 117.25, 119.24, 135.12, 153.18 ppm. EI-MS: 549.8 $[M]^+$.

$Cl_3PhO(CH_2)_{10}Br$ (Compound 14). ^{1}H-NMR (300 MHz, $CDCl_3$): δ = 1.23-1.44 (m, 10H, -*$(CH_2)_5$*-$(CH_2)_2$Br), 1.48-1.57 (m, 2H, -*CH_2*-$(CH_2)_7$Br), 1.78-1.89 (m, 4H, -*CH_2*-$(CH_2)_8$Br, -*CH_2*-CH_2Br), 3.37-3.42 (t, 2H, -*CH_2*Br), 3.95-4.00 (t, 2H, -*CH_2*-$(CH_2)_9$Br), 7.28 (s, 2H) ppm. ^{13}C-NMR (75 MHz, $CDCl_3$): δ = 25.73, 28.12, 28.69, 29.26, 29.30, 29.37, 29.95, 32.79, 33.84, 73.90, 128.65, 129.11, 130.08, 150.75 ppm.EI-MS: 416.0 $[M]^+$.

$I_3PhO(CH_2)_{10}Br$ (Compound 19). ^{1}H-NMR (300 MHz, $CDCl_3$): δ = 1.23-1.40 (m, 10H, -*$(CH_2)_5$*-$(CH_2)_2$Br), 1.48-1.55 (m, 2H, -*CH_2*-$(CH_2)_7$Br), 1.80-1.93 (m, 4H, -*CH_2*-$(CH_2)_8$Br, -*CH_2*-CH_2Br), 3.38-3.42 (t, 2H, -*CH_2*Br), 3.90-3.94 (t, 2H, -*CH_2*-$(CH_2)_9$Br), 8.03 (s, 2H) ppm. ^{13}C-NMR (75 MHz, $CDCl_3$): δ = 25.85, 28.11, 28.70, 29.33, 29.37, 29.64, 29.92, 32.78, 33.96, 73.51, 76.59, 88.86, 92.14, 147.17, 158.23 ppm. EI-MS: 689.9 $[M]^+$.

Synthesis of $Br_5PhO(CH_2)_{10}Br$ (Compound 24). ^{1}H-NMR (300 MHz, $CDCl_3$): δ = 1.33-1.52 (m, 12H, -*$(CH_2)_6$*-CH_2CH_2Br), 1.87 (m, 4H,-*CH_2*-CH_2Br and –OCH_2-*CH_2*-), 3.41 (t, 2H, J = 6.9 Hz, -*CH_2*Br), 3.99 (t, 2H, J = 6.3 Hz, -O-*CH_2*-). ^{13}C-NMR (75 MHz, $CDCl_3$): δ = 25.92, 28.30, 28.88, 29.50, 29.55, 30.00, 32.98, 34.08, 73.79, 122.03, 124.58, 128.49, 154.75 ppm. EI-MS: 707.8 $[M]^+$.

Synthesis of $PhO(CH_2)_{10}SH$ (Compound 2). The conversion of $PhO(CH_2)_{10}Br$ to $PhO(CH_2)_{10}SH$ followed the published procedure.[2] Into an oven-dried two-neck flask, 1.00 g (3.19 mmol) $PhO(CH_2)_{10}Br$ and 4.85 g (63.8 mmol) thiourea were suspended in ethanol under N_2. The mixture was refluxed for 3h, then aqueous KOH (63.8 mL, 1.0 M) was added and stirred for 1h. The mixture was then cooled down and ethanol was removed. The residual was dissolved in DCM and washed with deionized water for 3 times. The organic layers were combined, dried, filtered, and solvent was removed over evaporation. We purified the product

with column chromatography with hexane as eluent (yield 90 %). ^{1}H-NMR (300 MHz, $CDCl_3$): δ = 1.37-1.52 (m, 12H, -*$(CH_2)_6$*-CH_2CH_2SH), 1.67 (p, 2H, *J* = 6.3 Hz, -*CH_2*-CH_2SH), 1.84 (p, 2H, *J* = 6.9 Hz, -*CH_2*-$(CH_2)_8SH$), 2.58 (q, 2H, *J* = 6.3 Hz, -*CH_2*SH), 4.01 (t, 2H, *J* = 6.9 Hz, -O-*CH_2*-), 6.98 (m, 3H, *J* = 8.0 Hz, ArH), 7.33 (t, 2H, *J* = 8.0 Hz, ArH) ppm. ^{13}C-NMR (75 MHz, $CDCl_3$): δ = 24.71, 26.15, 28.45, 29.14, 29.40, 29.45, 29.526, 29.58, 34.13, 67.93, 114.59, 120.53, 129.46, 159.23 ppm. EI-MS: 266.2 $[M]^+$.**The synthesis procedures of Compounds 7, 8, 9, 10, 12, 16, 17, 18, and 20 are similar to Compound 2.**

FPhO$(CH_2)_{10}$SH (Compound 7). ^{1}H-NMR (300 MHz, $CDCl_3$): δ = 1.31-1.44 (m, 12H, -*$(CH_2)_6$*-CH_2CH_2SH), 1.61 (p, 2H, *J* = 6.6 Hz, -*CH_2*-CH_2-SH-), 1.76 (p, 2H, *J* = 7.5 Hz, -OCH_2-*CH_2*-), 2.52 (q, 2H, *J* = 7.5 Hz, -*CH_2*SH), 3.91 (t, 2H, *J* = 6.6 Hz, -O-*CH_2*-), 6.82 (m, 2H, ArH), 6.96 (m, 2H, ArH). ^{13}C-NMR (75 MHz, $CDCl_3$): δ = 24.77, 26.16, 28.50, 29.19, 29.43, 29.48, 29.56, 29.61, 36.17, 68.80, 115.53, 115.64, 115.69, 115.99, 155.41, 155.44, 155.70, 158.85 ppm. EI-MS: 283.9 $[M]^+$.

ClPhO$(CH_2)_{10}$SH (Compound 8). ^{1}H-NMR (300 MHz, $CDCl_3$): δ = 1.31-1.44 (m, 12H, -*$(CH_2)_6$*-CH_2CH_2SH), 1.61 (p, 2H, *J* = 6.6 Hz, -*CH_2*-CH_2-SH),1.76 (p, 2H, *J* = 7.2 Hz, -OCH_2-*CH_2*-), 2.52 (q, 2H, *J* = 7.2 Hz, -*CH_2*SH), 3.91 (t, 2H, *J* = 6.6 Hz, -O-*CH_2*-), 6.81 (m, 2H, ArH), 7.22 (m, 2H, ArH). ^{13}C-NMR (75 MHz, $CDCl_3$): δ = 24.76, 26.11, 28.48, 29.17, 29.32, 29.45, 29.54, 29.59, 34.15, 68.44, 115.90, 125.41, 129.37, 157.89 ppm. EI-MS: 299.9 $[M]^+$.

BrPhO$(CH_2)_{10}$SH (Compound 9). ^{1}H-NMR (300 MHz, $CDCl_3$): δ = 1.31-1.41 (m, 12H, -*$(CH_2)_6$*-CH_2CH_2SH), 1.61 (p, 2H, *J* = 7.2 Hz, -*CH_2*-CH_2SH), 1.76 (p, 2H, *J* = 6.6 Hz, -OCH_2-*CH_2*-), 2.52 (q, 2H, *J* = 7.2 Hz, -*CH_2*SH), 3.91 (t, 2H, *J* = 6.6 Hz, -O-*CH_2*-), 6.77 (d, 2H, *J* = 9.0 Hz, ArH), 7.36 (d, 2H, *J* = 9.0 Hz, ArH). ^{13}C-NMR (75 MHz, $CDCl_3$): δ = 24.78, 26.10, 28.48, 29.17, 29.28, 29.45, 29.54, 29.58, 34.15, 68.35, 112.69, 116.41, 132.30, 158.36 ppm. EI-MS: 343.9 $[M]^+$.

$IPhO(CH_2)_{10}SH$ (Compound 10). ^{1}H-NMR (300 MHz, $CDCl_3$): δ = 1.30-1.43 (m, 12H, -*$(CH_2)_6$*-CH_2CH_2SH), 1.61 (p, 2H, J = 6.6 Hz, -*CH_2*-CH_2-SH),1.76 (p, 2H, J = 7.2 Hz, -OCH_2-*CH_2*-), 2.52 (q, 2H, J = 7.2 Hz, -*CH_2*SH), 3.90 (t, 2H, J = 6.6 Hz, -O-*CH_2*-), 6.67 (m, 2H, ArH), 7.54 (m, 2H, ArH). ^{13}C-NMR (75 MHz, $CDCl_3$): δ = 24.78, 26.12, 28.49, 29.18, 29.28, 29.45, 29.55, 29.59, 34.16, 68.26, 83.54, 117.10, 138.29, 159.17 ppm. EI-MS: 392.0 $[M]^+$.

$Br_2PhO(CH_2)_{10}SH$ (Compound 12). ^{1}H-NMR (300 MHz, $CDCl_3$): δ = 1.31-1.41 (m, 12H, -*$(CH_2)_6$*-CH_2CH_2SH), 1.61 (p, 2H, J = 7.2 Hz, -*CH_2*-CH_2SH), 1.76 (p, 2H, J = 6.6 Hz, -OCH_2-*CH_2*-), 2.52 (q, 2H, J = 7.2 Hz, -*CH_2*SH), 3.91 (t, 2H, J = 6.6 Hz, -O-*CH_2*-), 6.98 (d, 2H, ArH), 7.22 (t, 1H, ArH). ^{13}C-NMR (75 MHz, $CDCl_3$): δ = 24.76, 26.05, 28.50, 29.15, 29.39, 29.55, 29.57, 34.16, 68.86, 117.20, 123.24, 126.40, 160.59 ppm. EI-MS: 422$[M]^+$.

$F_3PhO(CH_2)_{10}SH$ (Compound 16). ^{1}H-NMR (300 MHz, $CDCl_3$): δ = 1.29-1.46 (m, 12H, -*$(CH_2)_6$*-CH_2CH_2SH), 1.57-1.64 (m, 2H, -*CH_2*-CH_2SH), 1.68-1.77 (m, 2H, -*CH_2*-$(CH_2)_8SH$), 2.45-2.54 (q, 2H, -*CH_2*SH), 4.01-4.06 (t, 2H, -*CH_2*-$(CH_2)_9SH$), 6.62-6.68 (m, 2H, ArH) ppm. ^{13}C-NMR (75 MHz, $CDCl_3$): δ = 24.69, 25.71, 28.46, 29.14, 29.35, 29.51, 29.54, 29.97, 34.15, 75.22, 100.17, 100.30, 100.40, 100.53, 100.65, 100.75, 100.88, 132.60, 132.67, 132.79, 132.87, 132.99, 133.04, 154.53, 154.64, 154.73, 154.84, 155.25, 155.44, 155.63, 157.84, 157.94, 158.04, 158.14, 158.50, 158.69, 158.88 ppm. EI-MS: 320.2 $[M]^+$.

$Cl_3PhO(CH_2)_{10}SH$ (Compound 17). ^{1}H-NMR (300 MHz, $CDCl_3$): δ = 1.25-1.40 (m, 12H, -*$(CH_2)_6$*-CH_2CH_2SH), 1.64-1.68 (m, 2H, -*CH_2*-CH_2SH), 1.81-1.90 (m, 2H, -*CH_2*-$(CH_2)_8SH$), 2.51-2.59 (q, 2H, -*CH_2*SH), 3.99-4.03 (t, 2H, -*CH_2*-$(CH_2)_9SH$), 7.317 (s, 2H) ppm. ^{13}C-NMR (75 MHz, $CDCl_3$): δ = 24.55, 25.70, 28.28, 28.97, 29.26, 29.39, 29.93, 33.97, 73.85, 76.558, 76.98, 77.41, 128.60, 129.06, 130.04, 150.71 ppm. EI-MS: 368.0. $[M]^+$.

$Br_3PhO(CH_2)_{10}SH$ (Compound 18). ^{1}H-NMR (300 MHz, $CDCl_3$): δ = 1.31-1.61 (m, 14H, -*$(CH_2)_7$*-CH_2CH_2SH), 1.86 (p, 2H, J = 6.6 Hz,-OCH_2-*CH_2*-), 2.52 (q, 2H, J = 7.2 Hz, -*CH_2*SH),

3.97 (t, 2H, J = 6.6 Hz, -O-CH_2-), 7.74 (s, 2H, ArH). ^{13}C-NMR (75 MHz, $CDCl_3$): δ = 24.80, 25.96, 28.50, 29.19, 29.51, 29.59, 29.61, 30.10, 34.18, 73.87, 117.24, 119.24, 135.12, 153.20 ppm. EI-MS: 499.9 $[M]^+$.

$I_3PhO(CH_2)_{10}SH$ (Compound 20). ^{1}H-NMR (300 MHz, $CDCl_3$): δ = (m, 12H, -$(CH_2)_6$-CH_2CH_2SH), 1.53-1.66 (m, 2H, -CH_2-CH_2SH), 1.84-1.94 (m, 2H, -CH_2-$(CH_2)_8SH$), 2.48-2.56 (q, 2H, -CH_2SH), 3.91-3.95 (t, 2H, -CH_2-$(CH_2)_9SH$), 8.07 (s, 2H) ppm. ^{13}C-NMR (75 MHz, $CDCl_3$): δ = 25.85, 28.11, 28.70, 29.33, 29.64, 29.92, 32.78, 33.96, 73.52, 76.59, 77.02, 77.22, 77.44, 88.86, 92.14, 147.17, 158.23 ppm. EI-MS: 643.9 $[M]^+$.

Synthesis of $Br_5PhO(CH_2)_{10}SCOCH_3$ (Compound 25). We used another method to obtain the final thiol compound. First, we convert $(Br)_5PhO(CH_2)_{10}Br$ to $(Br)_5PhO(CH_2)_{10}SCOCH_3$. To a two neck flask, we added 0.71 g (1.0 mmol) $(Br)_5PhO(CH_2)_{10}Br$, 0.14 g (1.2 mmol), and 100 mL acetone. The mixture was refluxed for 1h, cooled down to room temperature. The acetone was removed and the residual was dissolved with DCM and washed with water. After combining the organic layers, they were dried over anhydrous $NaSO_4$, filtered the solid, and the filtrate was concentrated under reduced vacuum. The crude yield of this step was almost 100%, no column was needed. ^{1}H-NMR (300 MHz, $CDCl_3$): δ = 1.31-1.57 (m, 14H, -$(CH_2)_7$-$CH_2CH_2SCOCH_3$), 1.87 (p, 2H, J = 6.9 Hz, -CH_2-CH_2SCOCH_3), 2.32 (s, 3H, -SCOCH_3), 2.86 (t, 2H, J = 6.9 Hz, -CH_2SCO-), 3.99 (t, 2H, J = 6.3 Hz, -O-CH_2-). ^{13}C-NMR (75 MHz, $CDCl_3$): δ = 25.92, 28.94, 29.21, 29.31, 29.49, 29.53, 29.64, 30.00, 30.76, 73.82, 122.05, 124.57, 128.50, 154.79, 196.08 (-S*C*OCH$_3$) ppm. EI-MS: 701.9 $[M]^+$.

$F_5PhO(CH_2)_{10}SCOCH_3$ (Compound 22). Compound 22 was synthesized using the same procedure as Compound 25. ^{1}H-NMR (300 MHz, $CDCl_3$): δ = 1.29-1.56 (m, 14H, -$(CH_2)_7$-$(CH_2)_2SCOCH_3$), 1.75 (p, 2H, J= 7.2 Hz, -CH_2-CH_2SCOCH_3), 2.31 (s, 3H, SCOCH_3), 2.86 (t, 2H, J= 7.2 Hz, -CH_2SCOCH_3), 4.14 (t, 2H, J= 6.6 Hz, -CH_2-$(CH_2)_9SCOCH_3$) ppm. ^{13}C-NMR

(75 MHz, $CDCl_3$): δ = 25.64, 28.93, 29.19, 29.29, 29.47, 29.52, 29.65, 29.97, 30.71, 75.98, 133.62, 133.64, 133.66, 133.74, 133.76, 133.79, 133.87, 133.76, 133.79, 133.87, 133.90, 133.94, 135.86, 135.90, 135.92, 135.99, 136.03, 136.07, 136.66, 136.68, 136.74, 136.81, 136.85, 136.89, 138.21, 138.24, 138.31, 138.33, 138.41, 138.45, 139.01, 139.06, 139.10, 139.16, 139.20, 139.24, 139.27, 139.33, 139.38, 140.47, 140.51, 140.64, 140.67, 142.98, 143.03, 143.07, 143.10, 143.14, 143.18, 196.06 ppm.EI-MS: 398.2 $[M]^+$.

Synthesis of $Br_5PhO(CH_2)_{10}SH$ (Compound 26). We converted the $(Br)_5PhO(CH_2)_{10}SCOCH_3$ to $(Br)_5PhO(CH_2)_{10}SH$ following a previously reported method.[3] 0.50 g (0.71 mmol) $(Br)_5PhO(CH_2)_{10}SCOCH_3$ was dissolved in 50 mL chloroform and methanol (1:1) and added into a two-neck flask under N_2, followed by the addition of 0.11 g (0.355 mmol) tetrabutylammonium cynide (TBACN, a toxic compound, keep away from acids or acidic solutions). The mixture was stirred for 5 h before solvents were removed. The solid was dissolved with DCM and washed with water for 3 times. Organic layers were combined, dried, filtered and evaporated. The product was purified over column chromatography using hexane and DCM at 10: 1. Yield 0.18 g (38 %). ^{1}H-NMR (300 MHz, $CDCl_3$): δ = 1.31-1.61 (m, 14H, -$(CH_2)_7$-CH_2CH_2SH), 1.87 (p, 2H, J = 6.9 Hz, -CH_2-CH_2SH), 2.52 (q, 2H, J = 7.2 Hz, -CH_2SH), 3.99 (t, 2H, J = 6.3 Hz, -O-CH_2-). ^{13}C-NMR (75 MHz, $CDCl_3$): δ = 24.79, 25.94, 28.51, 29.20, 29.52, 29.60, 30.02, 34.18, 73.84, 122.05, 124.60, 128.51, 154.80 ppm. EI-MS: 659 $[M]^+$.

$F_5PhO(CH_2)_{10}SH$ (Compound 23). Compound 23 was synthesized using the same procedure as Compound 26. ^{1}H-NMR (300 MHz, $CDCl_3$): δ = 1.30-1.42 (m, 12H, -$(CH_2)_6$-$(CH_2)_2SH$), 1.61 (p, 2H, J= 6.6, Hz-CH_2-$(CH_2)_8SH$), 1.76 (p, 2H, J= 7.2 Hz, -CH_2-CH_2SH), 2.52 (q, 2H, J= 7.2 Hz, -CH_2SH), 4.14 (t, 2H, J= 6.6 Hz, -CH_2-$(CH_2)_9SH$) ppm. ^{13}C-NMR (75 MHz, $CDCl_3$): δ = 24.76, 25.65, 28.50, 29.17, 29.33, 29.53, 29.55, 29.98, 34.18, 75.98, 133.65, 133.69, 133.77, 133.82, 133.90, 133.94, 135.82, 135.89, 135.93, 135.95, 136.02,

136.06, 136.10, 136.48, 136.50, 136.53, 136.69, 136.71, 136.77, 136.83, 136.88, 136.92, 138.23, 138.26, 138.31, 138.34, 138.37, 138.44, 138.46, 138.59, 138.52, 138.57, 139.04, 139.08, 139.12, 139.19, 139.23, 139.28, 139.36, 139.41, 139.48, 140.50, 140.55, 140.59, 140.62, 140.64, 140.66, 140.70, 140.71, 140.75, 142.97, 143.01, 143.08, 143.11, 143.13, 143.17, 143.21 ppm.EI-MS: 356.0 $[M]^+$.

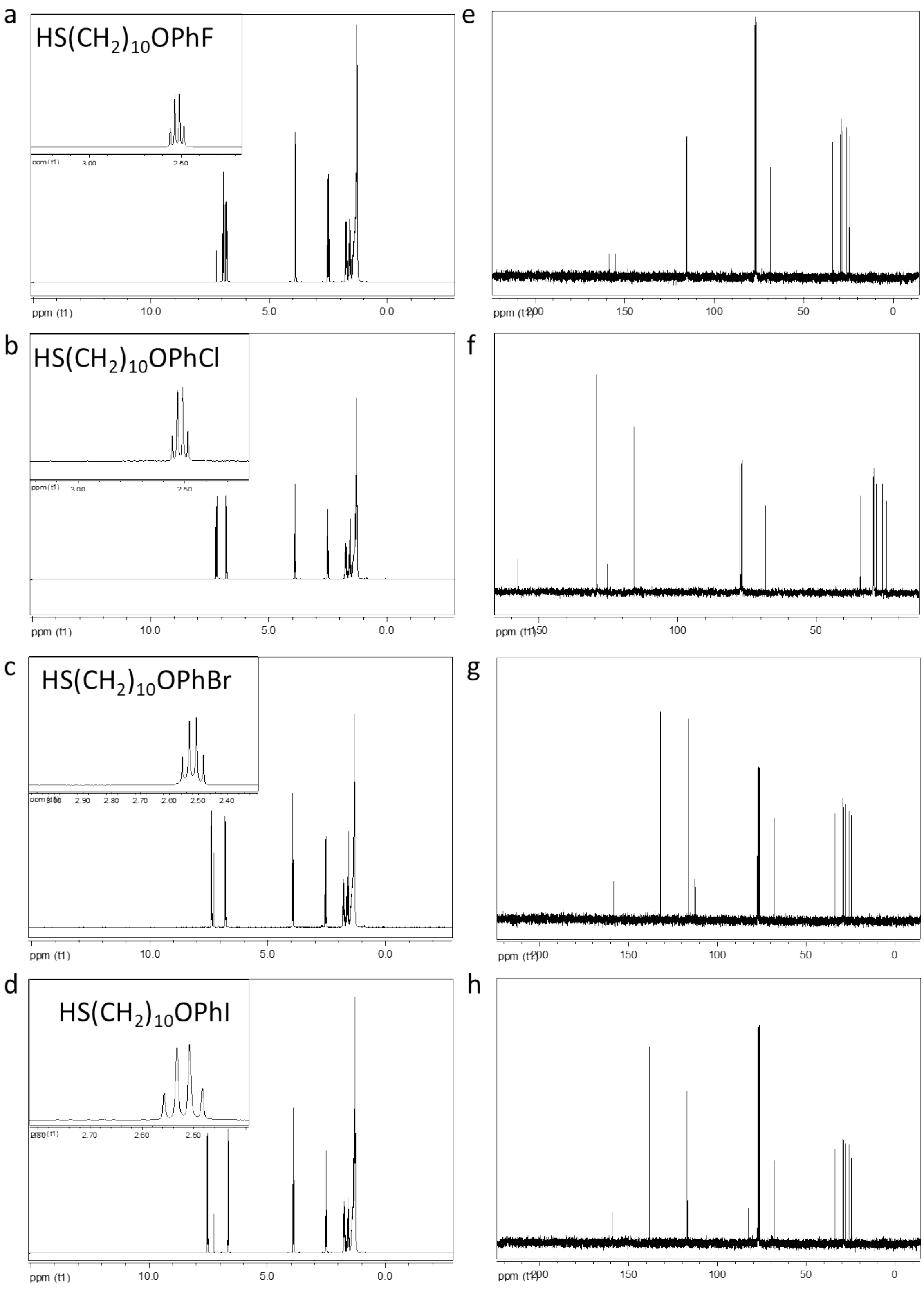

Figure S2. H[1] NMR spectra of $HS(CH_2)_{10}OPhX$ with X = F, Cl, Br, and I shown in a-d respectively.^{13}C NMR spectra $HS(CH_2)_{10}OPhX$ molecules with X = F, Cl, Br, and I shown in e-h respectively.

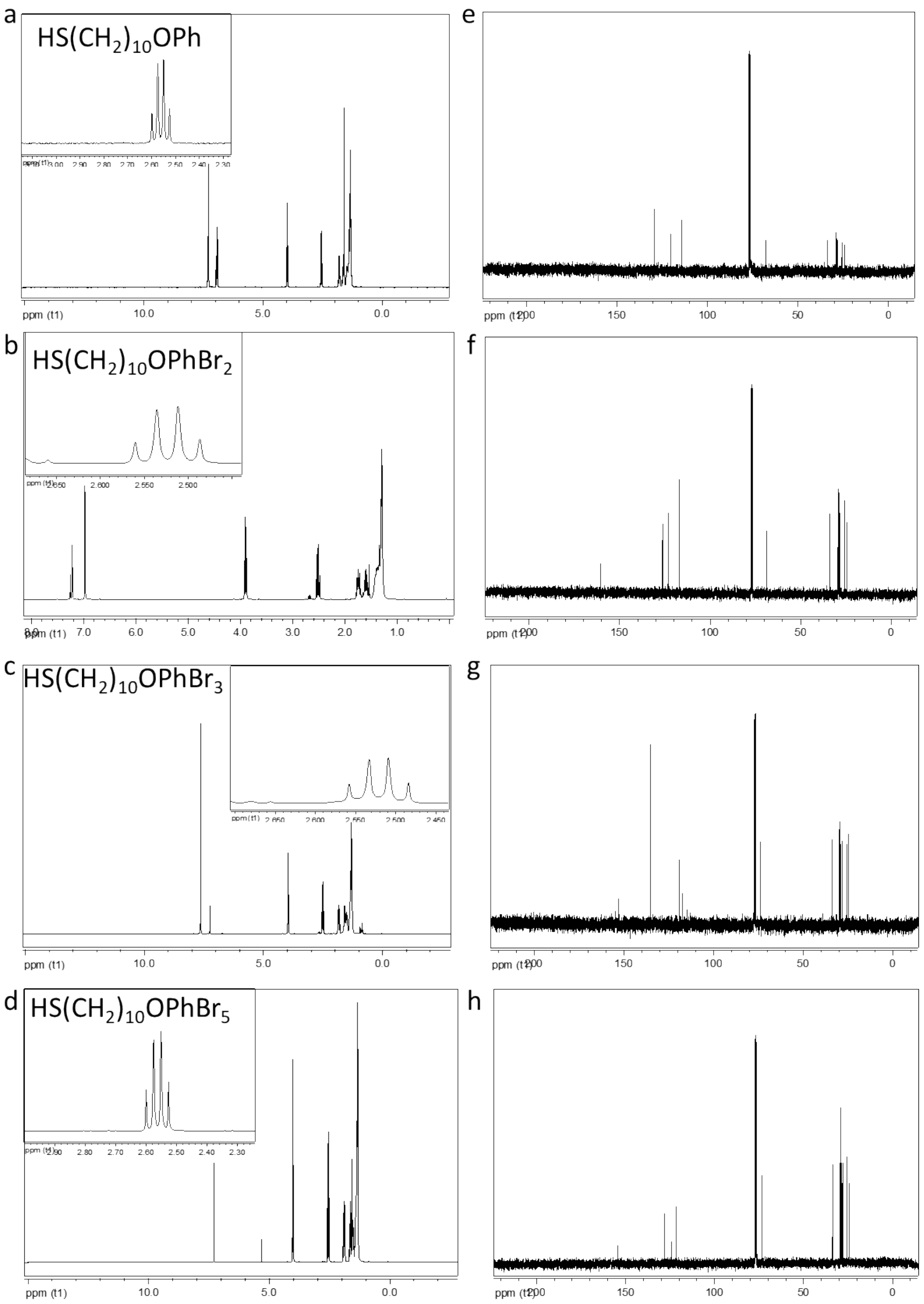

Figure S3. H^1 NMR spectra of $HS(CH_2)_{10}OPhBr_n$ with $n = 0$, 2, 3, and 5shown in a-d respectively. ^{13}C NMR spectra $HS(CH_2)_{10}OPhBr_n$ with $n = 0$, 2, 3, and 5 in e-h respectively.

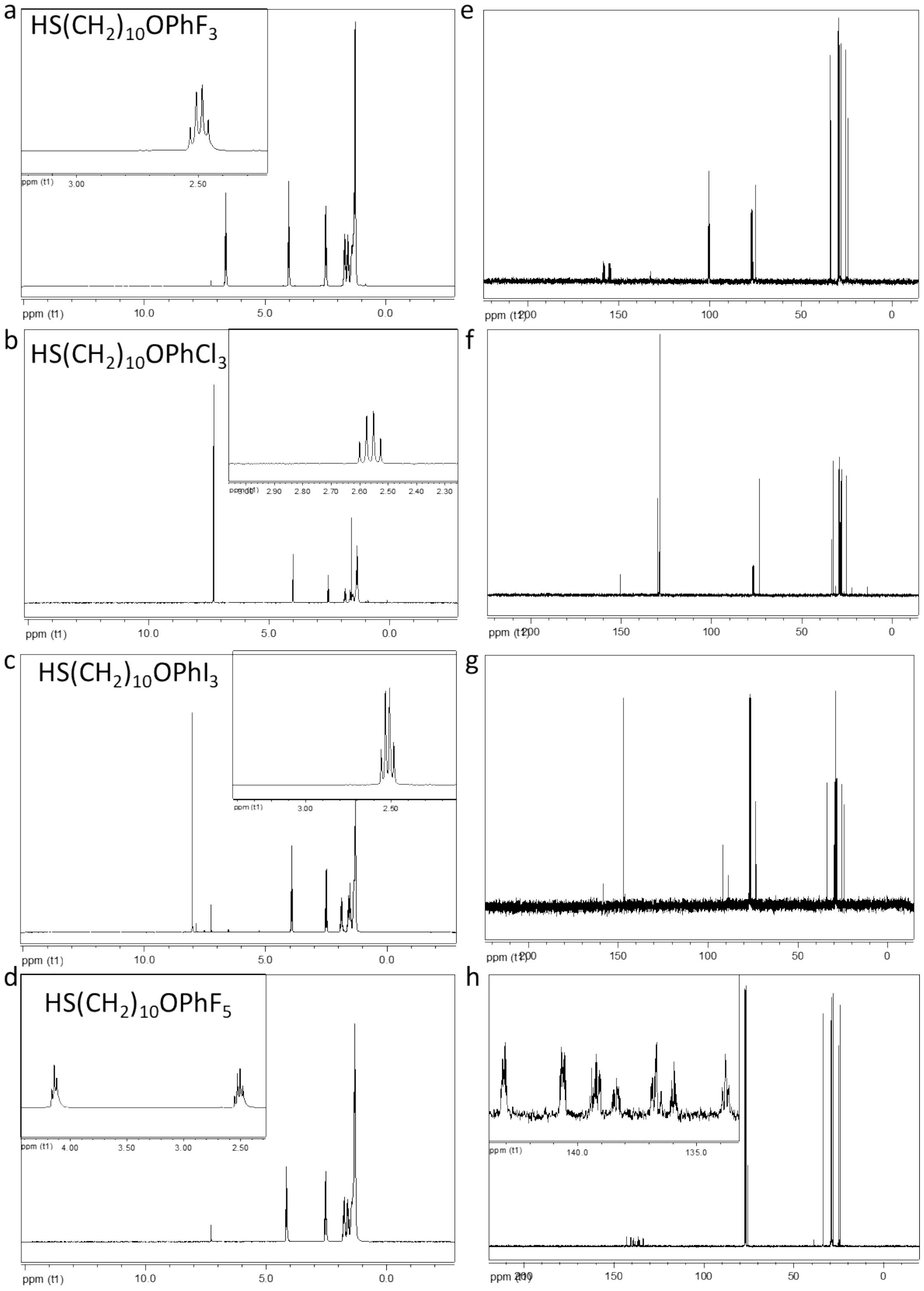

Figure S4. H^1 NMR spectra of $HS(CH_2)_{10}OPhX_3$ with X = F, Cl, and I shown in a-c respectively, and $HS(CH_2)_{10}OPhBr_5$(d). ^{13}C NMR spectra of $HS(CH_2)_{10}OPhX_3$ with X = F, Cl, and I shown in panels e-g respectively, and $HS(CH_2)_{10}OPhBr_5$ (h).

Section S2: Preparation of Ag substrates and SAMs

Preparation of Ag. The deposition and annealing procedures of Ag using thermal evaporator, and the preparation of template-stripped Ag by gluing glass slides were the same as previous report.[4]We used Bruker Dimension Fastscan AFM (FASTSCAN-A) in tapping mode to determine the surface roughness of Ag. The average rms roughness was 0.5 ± 0.1 nmrecorded from substrates of 5 × 5 μm^2 (the error is the standard deviation from three separate measurements from three substrates).

SAM preparation. The SAM was prepared as follows. We dissolved a certain amount of monolayer precursor in an oven dried vial, added 5 mL freshly distilled ethanol to obtain 1 mM ethanolic solutions. A freshly template-stripped Ag surface was then immersed in the solution and kept under N_2 protection for overnight. The substrate was taken out and washed with ethanol and then dried under N_2 flow.

Section S3: SAM Characterization

All the synchrotron-based data were recorded at the SINS (Surface, Interface and Nanostructure Science) beamline of Singapore Synchrotron Light Source (SSLS) following reported method.[5] All samples were kept under ultrahigh vacuum of 10^{-9} mbar pressure throughout the experiment. The XPS data were fitted using XPS PEAK software v. 4.1 (Raymund Kwok, The Chinese University of Hong Kong, Shatin, Hong Kong).

X-ray photoelectron spectroscopy (XPS). We used synchrotron based soft X-ray beams to probe the surface structure of the Ag-$S(CH_2)_{10}OPhX_n$ SAMs (here we used series of X =Br and n = 3). Angle resolved X-ray photoelectron spectroscopy (ARXPS) was used to calculate the thickness of the SAM in nm (as shown in Table 1).[4, 6] The take-off (θ) angle defined as the angle between the detected beam and the substrate was altered between 90° (normal emission) and 40° (grazing incidence) by rotating the sample stage as angle between the analyser and the sample stage is fixed as 50°. The spectra for each n are summarised in Figures S5–S11. Figure S5 compares C1*s* spectra where the peak intensity corresponding to C=C-Br (~286.0eV) clearly shows increase with increasing n, while the peak intensity corresponding to C-C (~284.4 eV). Figure S6 compares S 2p peaks - S 2$p_{3/2}$ was located around (~161.8 eV) and a spin orbit splitting (SOS) of 1.18 eV was used to fit S 2$p_{1/2}$. We determined thickness of the SAM (d_{SAM}) from the S 2p ARXPS using Eqs. S1 and S2.[4, 6] I_θ at 90° was corrected with a cos50°. The free mean path λ was taken as 8 Å and Ag-S bond distance ($d_{Ag\text{-}S}$~1.8 Å) was added to d to get d_{SAM}.

$$I_\theta = I_0 e^{(-\frac{d}{\lambda})} \quad \text{(Eq. S1)}$$

$$d_{SAM} = d + d_{Ag-S} \text{(Eq. S2)}$$

Figure S7 compares Br 3*d*peaks for n = 1, 2, 3, 5 SAMs, Br 3$d_{5/2}$ is located at 70.5 eV and an SOS of 1.04 eV was used. Figure S9 shows the peaks for Ag 3*d* fitted with an SOS of 6.00 eV between Ag 3$d_{5/2}$ (~368.2 eV)and Ag 3$d_{5/2}$. The relative surface coverage (relative to n = 0 SAM) was calculated from the peak intensity of S2p at θ = 90°. For the relative coverage calculation, ratio of I_S/I_{Ag} were compared.

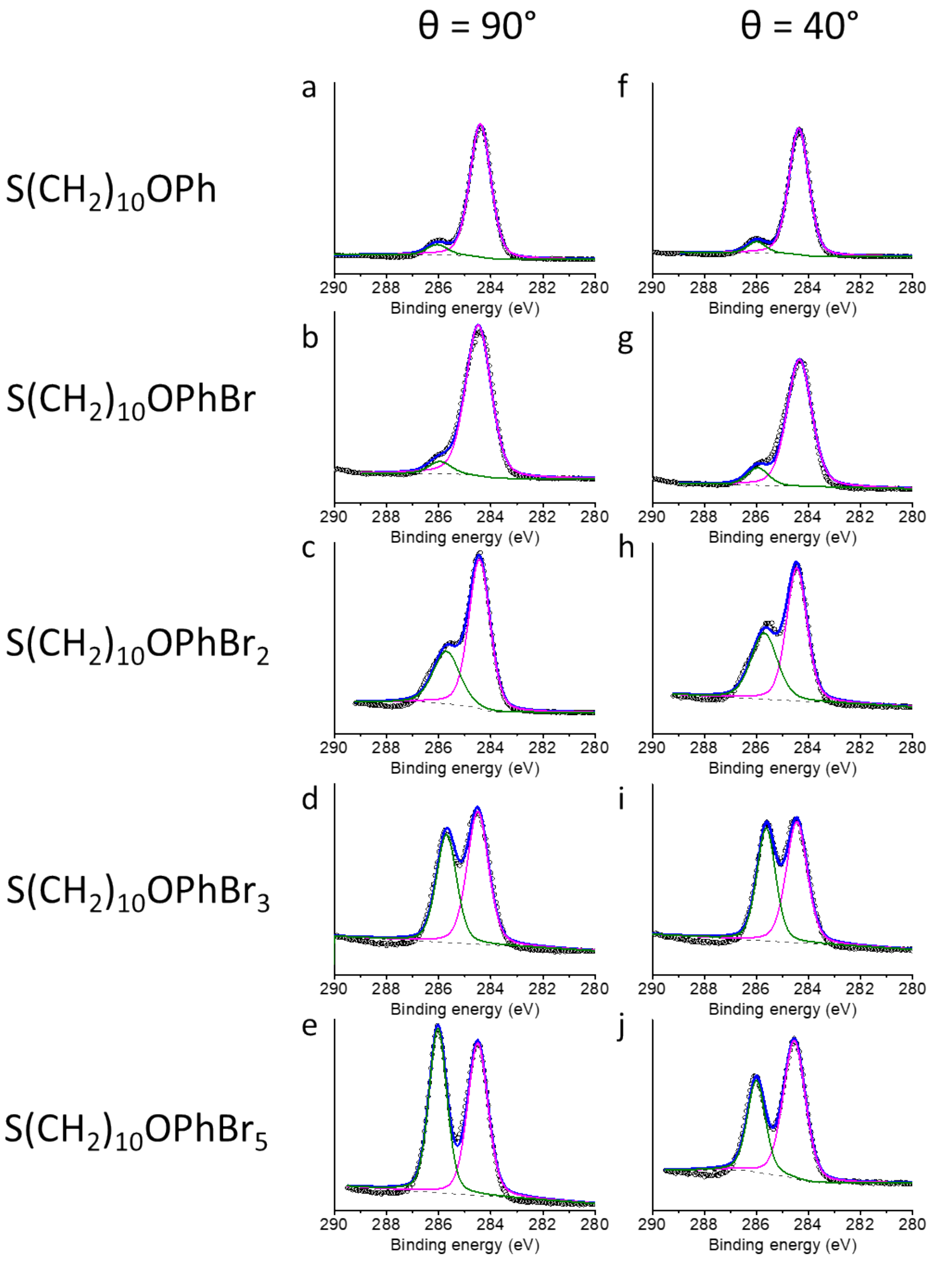

Figure S5. C1*s*XPS peaks of Ag-S$(CH_2)_{10}$OPhBr$_n$ SAMs where n = 0 (a, f), n = 1 (b, g), n = 2 (c, h), n = 3 (d, i), and n = 5(e, j) for two take-off angles θ as indicated.

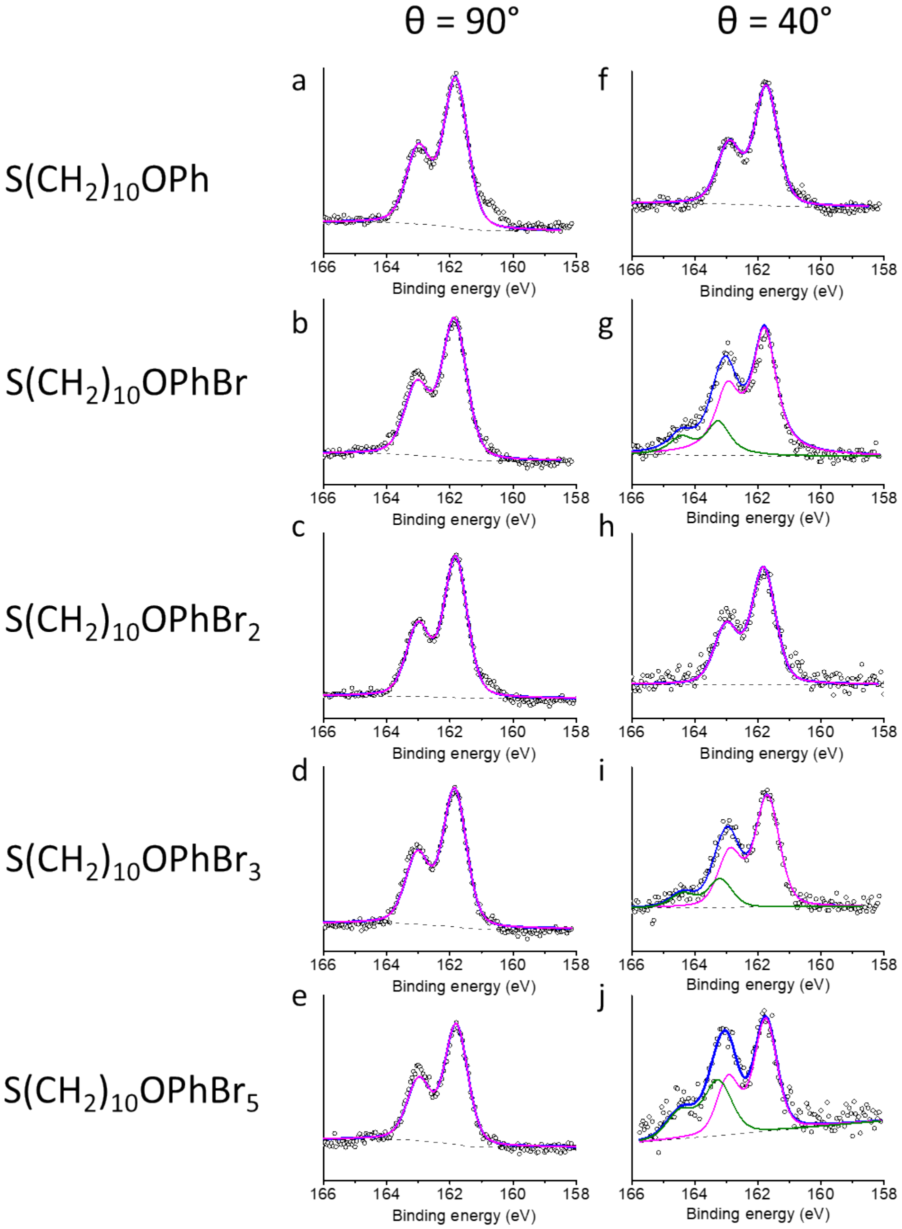

Figure S6. S2*p* XPS peaks of Ag-$S(CH_2)_{10}OPhBr_n$ SAMs where n = 0 (a, f), n = 1 (b, g), n = 2 (c, h), n = 3 (d, i), and n = 5(e, j) for two take-off angles θ as indicated.

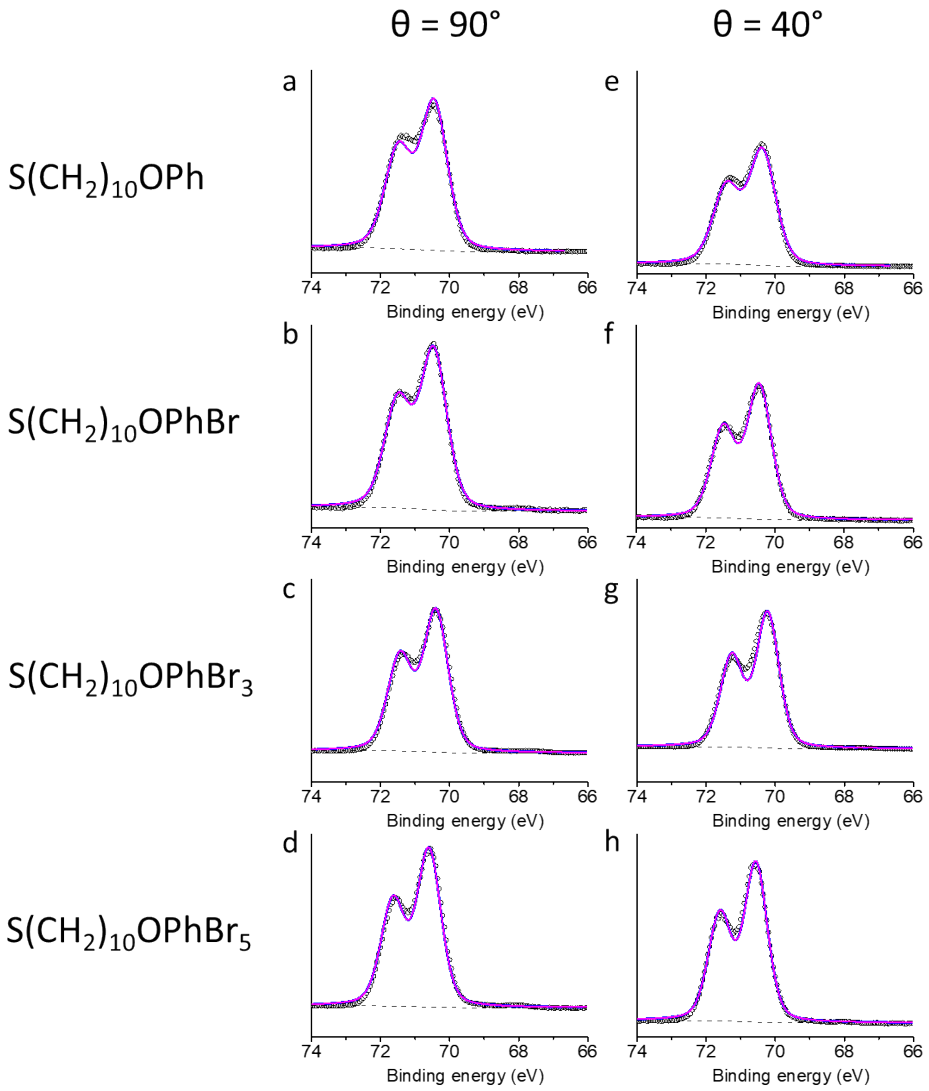

Figure S7. Br 3*d*XPS peaks of Ag-S$(CH_2)_{10}$OPhBr$_n$ SAMs where n = 0 (a, f), n = 1 (b, g), n = 2 (c, h), n = 3 (d, i), and n = 5(e, j) for two take-off angles θ as indicated

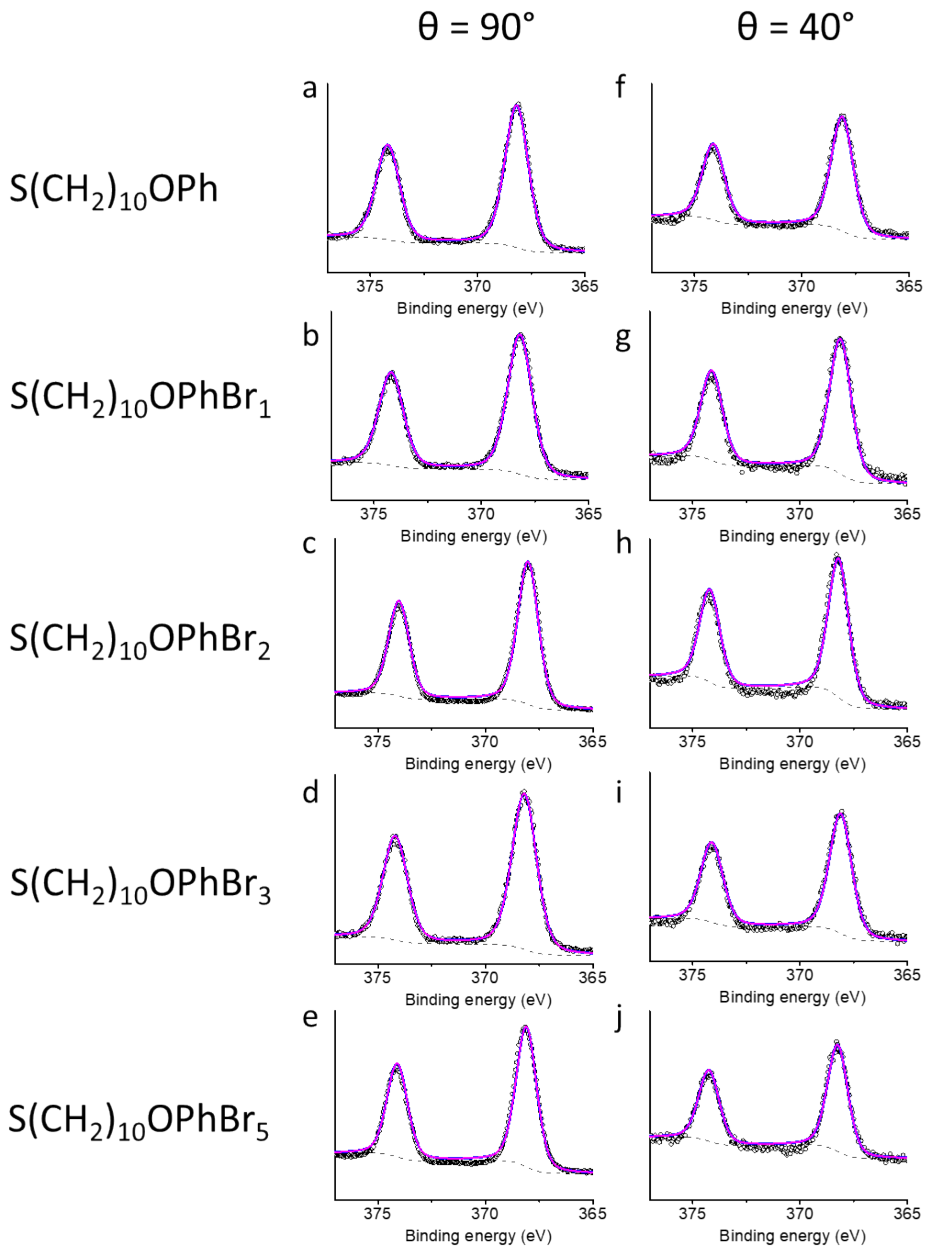

Figure S8. Ag 3*d* XPS peaks of Ag-$S(CH_2)_{10}OPhBr_n$ SAMs where $n = 0$ (a, f), $n = 1$ (b, g), $n = 2$ (c, h), $n = 3$ (d, i), and $n = 5$(e, j) for two take-off angles θ as indicated.

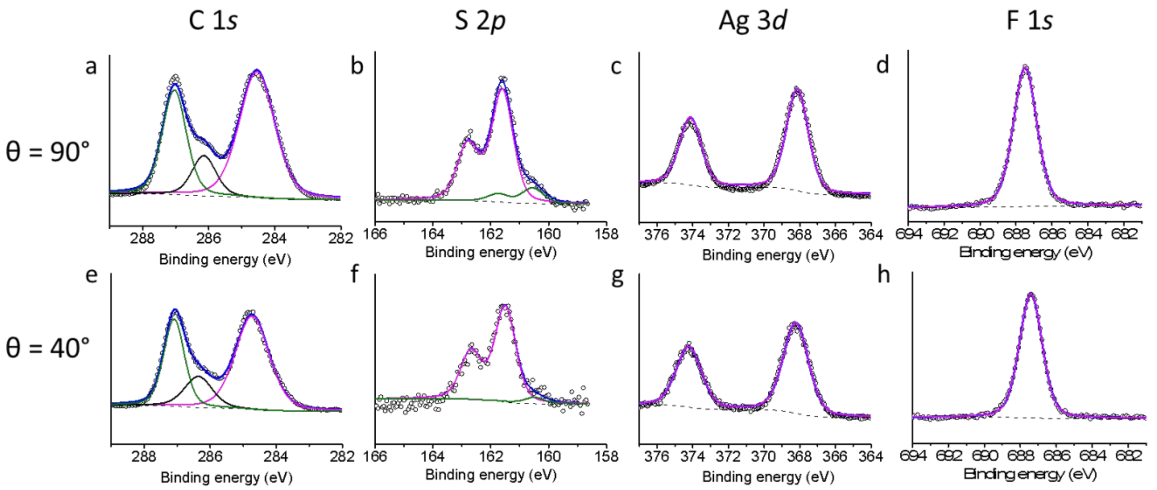

Figure S9. C 1*s*, S 2*p*, Ag 3*d*, and F 1*s* XPS peaks of Ag-S$(CH_2)_{10}$OPhF_3SAM for two take-off angles θ as indicated.

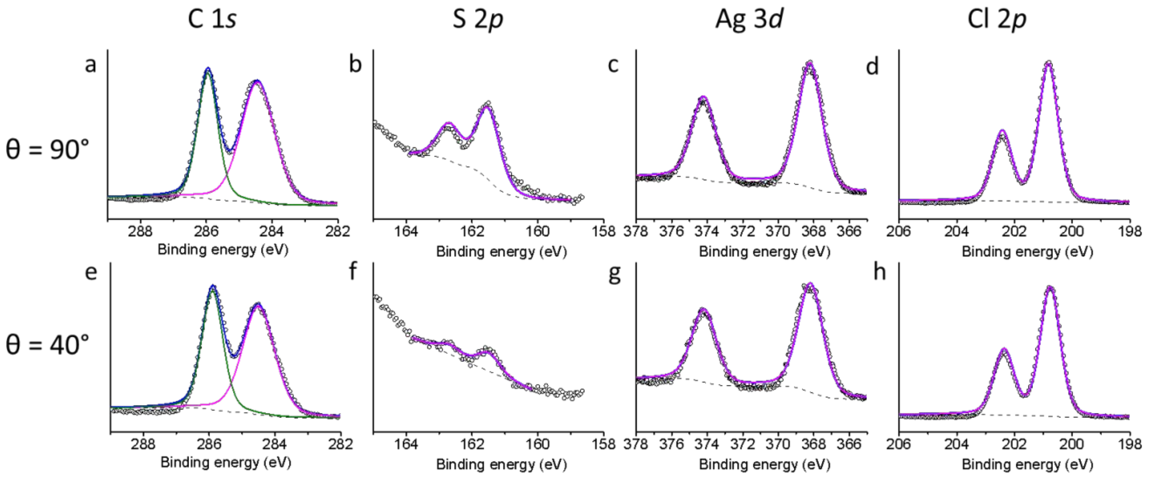

Figure S10. C 1*s*, S 2*p*, Ag 3*d*, and Cl 2*p*XPS peaks of Ag-S$(CH_2)_{10}$OPhCl_3SAM for two take-off angles θ as indicated.

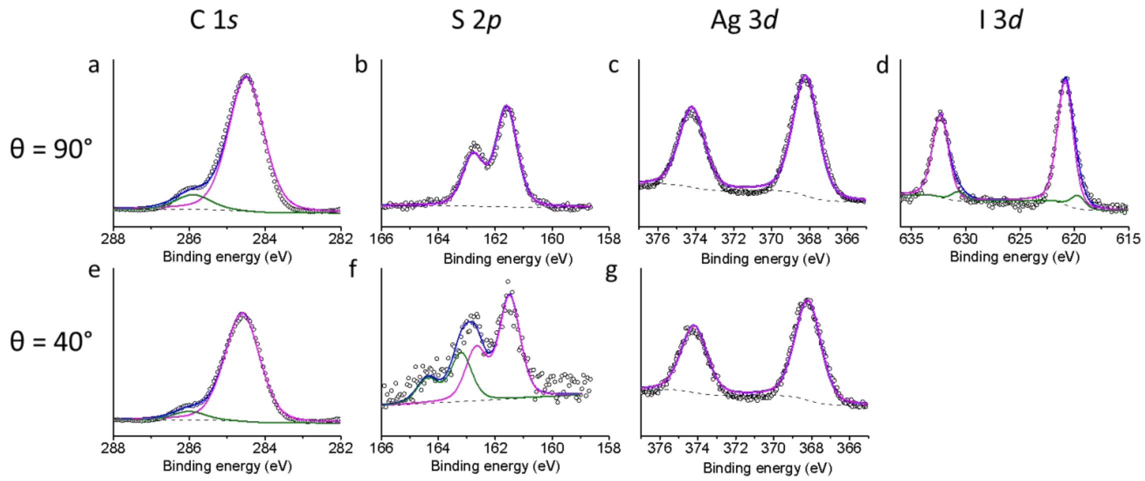

Figure S11. C 1*s*, S 2*p*, Ag 3*d*, and I 3*d*XPS peaks of Ag-S$(CH_2)_{10}$OPhI_3SAM for two take-off angles θ as indicated.

Ultraviolet photoelectron spectroscopy (UPS). The UPS data, summarised in Figure S12, was measured using a lab-based system. The source was a He Iα light and the analyser was a 7-channel detection system with an Omicron EA125 U7 hemispherical electron spectrometer installed on the VG Scientific ESCALab Mark 2 system. The secondary cut-off end was used to calculate work function and the valence band was used to estimate the HOMO onset.[4, 6] Together, they gave an estimate of the HOMO level.

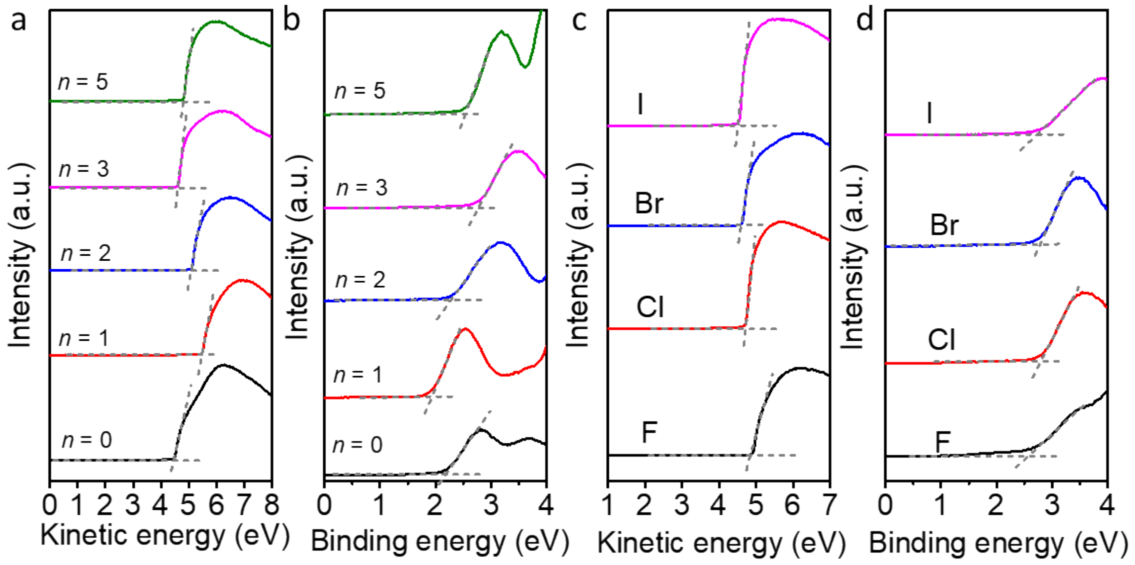

Figure S12. (a) Secondary electron cut-off spectra of and (b) valence band of Ag-$S(CH_2)_{10}OPhBr_n$ SAMsfrom UPS. The cross-section of the dashed lines indicates work function and HOMO onset. (**c**) Secondary electron cut-off spectra of and (d) valence band of Ag-$S(CH_2)_{10}OPhX_3$ SAMs from UPS.

Near edge X-ray absorption fine structure (NEXAFS). Figures S13 summarizes NEXAFS spectra recorded at the C K-edge to estimate the orientation of the aromatic head group following previously reported methods[5-6]. To calculate the average tilt angle of the aromatic head group with respect to the surface normal, the following formula, in which peak intensities ratio at 90° and 40° incident angles (note that these angles are different from θ) and the linear polarisation factor P = 0.90, was used. The peak intensities at ~285.1 eV corresponds to the C 1*s*of C-Hon phenyl ring and the peak at 286.3 eV is assigned to the C 1*s* of C-Br group. Figures S13 shows NEXAFS spectra recorded at the C K edge of NEXAFS spectra at 90° and 20° incidence angle. The decrease in C-H (on phenyl ring) intensity and increase in C-Br with the increasing value of *n* (Figure S13a) indicates the transition of C-H bonds to C-Br. The (C-X) shifts towards higher photon energy with increasing χ of X, as shown in Figure S13b, which agrees with the XPS data.

$$\frac{I_{90}}{I_{40}} = \frac{P\left[sin^2(90^o)sin^2(\alpha) + 2cos^2(90^o)cos^2(\alpha)\right] + (1-P)[sin^2(\alpha)]}{P[sin^2(40^o)sin^2(\alpha) + 2cos^2(40^o)cos^2(\alpha)] + (1-P)[sin^2(\alpha)]}$$

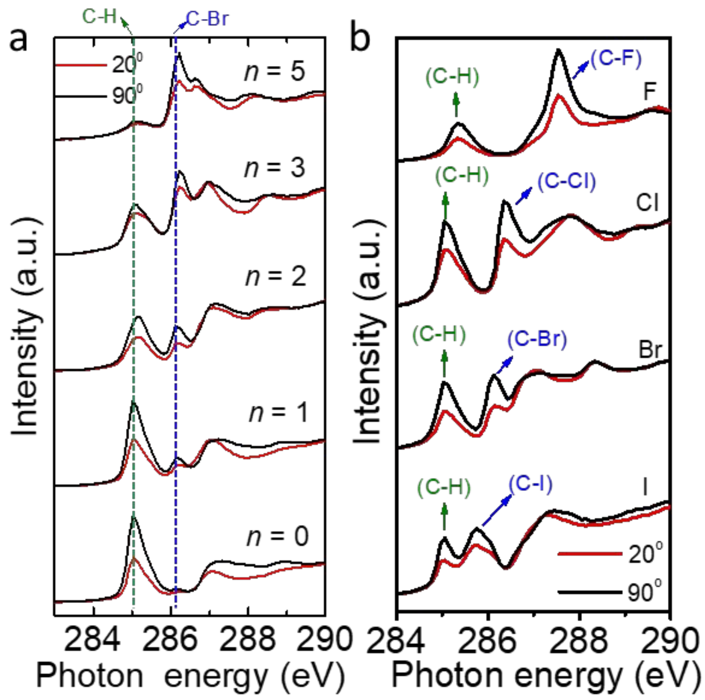

Figure S13. (a) C K edge NEXAFS of Ag-$S(CH_2)_{10}OPhBr_n$ SAMs and (b) Ag-$S(CH_2)_{10}OPhX_3$ SAMs at 90° (black lines) and 20° (red lines) incidence angle. The green dashed line indicates the C 1*s* signal of C-H on phenyl ring, the blue dashed line indicates the C-Br.

Table S1. Summary of spectroscopy data of Ag-$S(CH_2)_{10}OPhBr_n$ SAMs

n or X	γ (°)[a]	d_{SAM} (nm)[b]	Γ_{SAM}[b]	E_{HOMO} (eV)[c]	ΔE_{HOMO} (eV)[c]	Φ_{SAM} (eV)[c]
0	25.2	1.7	1.0	-6.66	2.21	-4.45
1	26.5	2.3	1.1	-7.42	1.95	-5.47
2	28.1	2.1	1.0	-7.42	2.35	-5.07
3	27.2	2.4	0.86	-7.40	2.80	-4.60
5	33.4	2.2	0.80	-7.35	2.55	-4.80

[a]Average tilt angle of the phenyl group was obtained from angular dependent C K-edge NEXAFS. Error = ± 5°.
[b]ARXPS was used to get film thickness and relative surface coverage (relative to Γ_{SAM} of $S(CH_2)_{10}OPh$ on Ag SAMs = 1.0 (error = 10% from instrumental and fit error for both d_{SAM} and Γ_{SAM}).
[c]UPS was used to get HOMO, HOMO offset and Φ_{SAM}. Resolution = ± 0.1 eV.

Table S2. Summary of spectroscopy data of Ag-$S(CH_2)_{10}OPhX_3$ SAMs

X	γ (°)[a]	d_{SAM} (nm)[b]	Γ_{SAM}[b]	E_{HOMO} (eV)[c]	ΔE_{HOMO} (eV)[c]	Φ_{SAM} (eV)[c]
H	25.2	1.7	1.0	-6.66	2.21	-4.45
F	28.4	1.7	1.0	-7.53	2.64	-4.89
Cl	28.9	2.0	0.82	-7.53	2.81	-4.72
Br	27.2	2.4	0.86	-7.40	2.80	-4.60
I	28.2	2.4	0.96	-7.18	2.73	-4.45

Section S4 Optical Gaps and DFT Calculations

The optical gaps ($\Delta E_{H\text{-}L,O}$) of the molecules were measured with UV-vis spectrometer (PerkinElmer LAMBDA 750 UV/vis/NIR spectrophotometer)[7] in dichloromethane solution and shown in Figures S14-16. The polarizabilities (*a*) of the molecules were calculated using the density functional theory (DFT) with Gaussian 09 Revision D.01 software using the B3LYP method and the CEP-31G**basis set on each molecule in the gas phase. From Table S3, when the halogen atom increases in size from H to I, or the number of Br increases, the*a* increases and this influences the *a* of the corresponding molecule to increase.

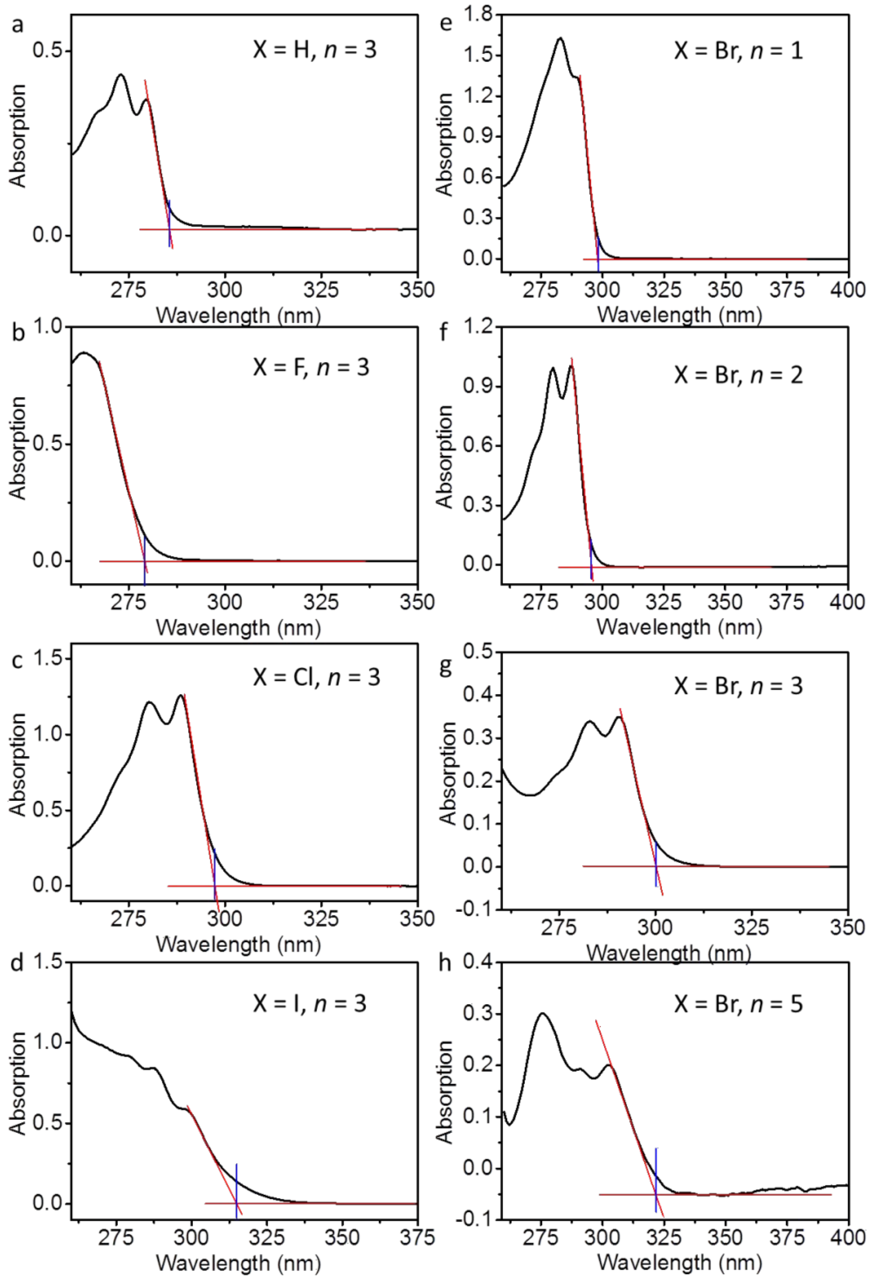

Figure S14. UV-visabsorption spectra of $HS(CH_2)_{10}OPhX_3$ and $HS(CH_2)_{10}OPhBr_n$ in DCM. The blue lines at the cross-section of the red lines are used to determine the $\Delta E_{H\text{-}L,O}$ of the molecules.

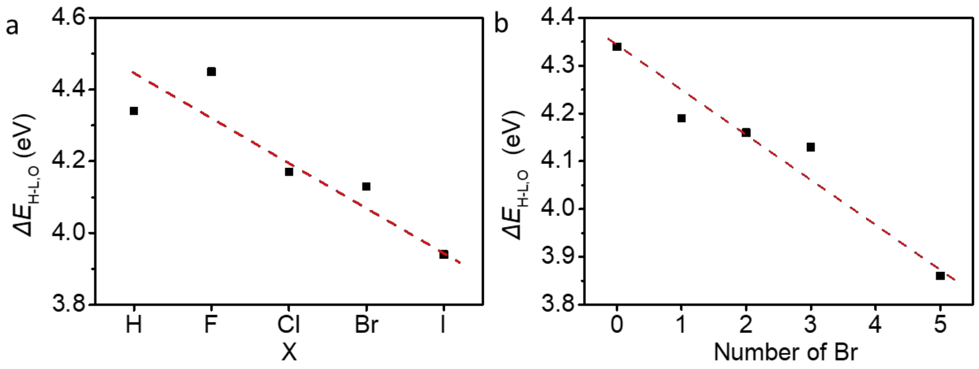

Figure S15. The optical HOMO-LUMP gaps,Δ$E_{\text{H-L,O}}$,of $HS(CH_2)_{10}OPhX_3$(a) and $HS(CH_2)_{10}OPhBr_n$(b) from UV-visabsorption spectra recorded in DCM.

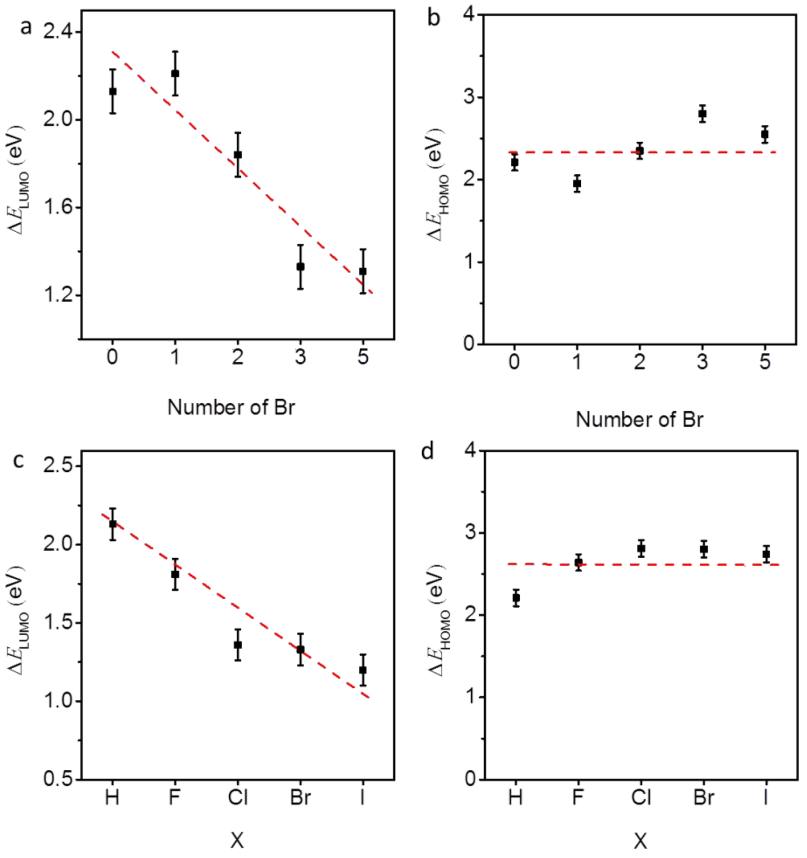

Figure S16. The ΔE_{LUMO} of $S(CH_2)_{10}OPhBr_n$and $S(CH_2)_{10}OPhX$ SAMs on Ag (a and c, respectively), and ΔE_{HOMO} of $S(CH_2)_{10}OPhBr_n$and $S(CH_2)_{10}OPhX_3$ SAMs on Ag (b and d, respectively).

Table S3. Summary $\Delta E_{H\text{-}L,O}$, E_{LUMO} and ΔE_{LUMO} of $HS(CH_2)_{10}OPhX_3$ and $HS(CH_2)_{10}OPhBr_n$

n and X	Absorption peak onset λ (nm)	$\Delta E_{H\text{-}L,O}$ (eV)	E_{LUMO} (eV)[d]	ΔE_{LUMO} (eV)[e]	a (Å^3)
n = 3, X = H	285	4.34	-2.32	2.13	30.8
n = 3, X = F	279	4.45	-3.08	1.81	30.7
n = 3, X = Cl	297	4.17	-3.36	1.36	36.3
n = 3, X = Br	300	4.13	-3.27	1.33	39.4
n = 3, X = I	315	3.94	-3.24	1.20	45.1
n = 1, X = Br	298	4.19	-3.23	2.21	33.2
n = 2, X = Br	296	4.16	-3.26	1.84	36.0
n = 5, X = Br	322	3.86	-3.49	1.31	44.2

[d]E_{LUMO} values were determined from the difference between E_{HOMO} and $\Delta E_{H\text{-}L,O}$;
[e]ΔE_{LUMO}values were calculated from offset between Φ_{SAM} and E_{LUMO}.

Section S5: Junction Characterization

Junction setup and data analysis. The collection of J(V) curves were done with an EGaIn setup as reported before.[4, 8] A keithley 6340 source meter and Labview 2010 were used to apply voltage and collect data. We collected about 20 junctions and for each junction about 20 scans for each type of sample. To thelogarithm current density, $\log_{10}|J|$, we fitted at each bias to obtain the Gaussian log-average, $\langle \log_{10}|J| \rangle_G$, with log-standard deviation of $\sigma_{log,G}$from ~400 J(V) curves. For the rectification ratio (R) calculation, we used a similar method. We first calculated the $\log_{10}R$ at ±1.0 V (the log values of$\frac{J(V)_{+1.0\text{ V}}}{J(V)_{-1.0\text{ V}}}$) for all the traces, then we fitted a Gaussian to the $\log_{10}R$histogram to obtain the Gaussian log-R, $\langle \log_{10}R \rangle_G$, and log-standard deviation of $\sigma_{log,G}$. Figure S17-23and Tables S4-6 show the $\langle \log_{10}|J| \rangle_G$ vs applied bias V for all the types of junctions and the histograms of the $\log_{10}R$ and $\log_{10}|J|$ at ±1.0 V.

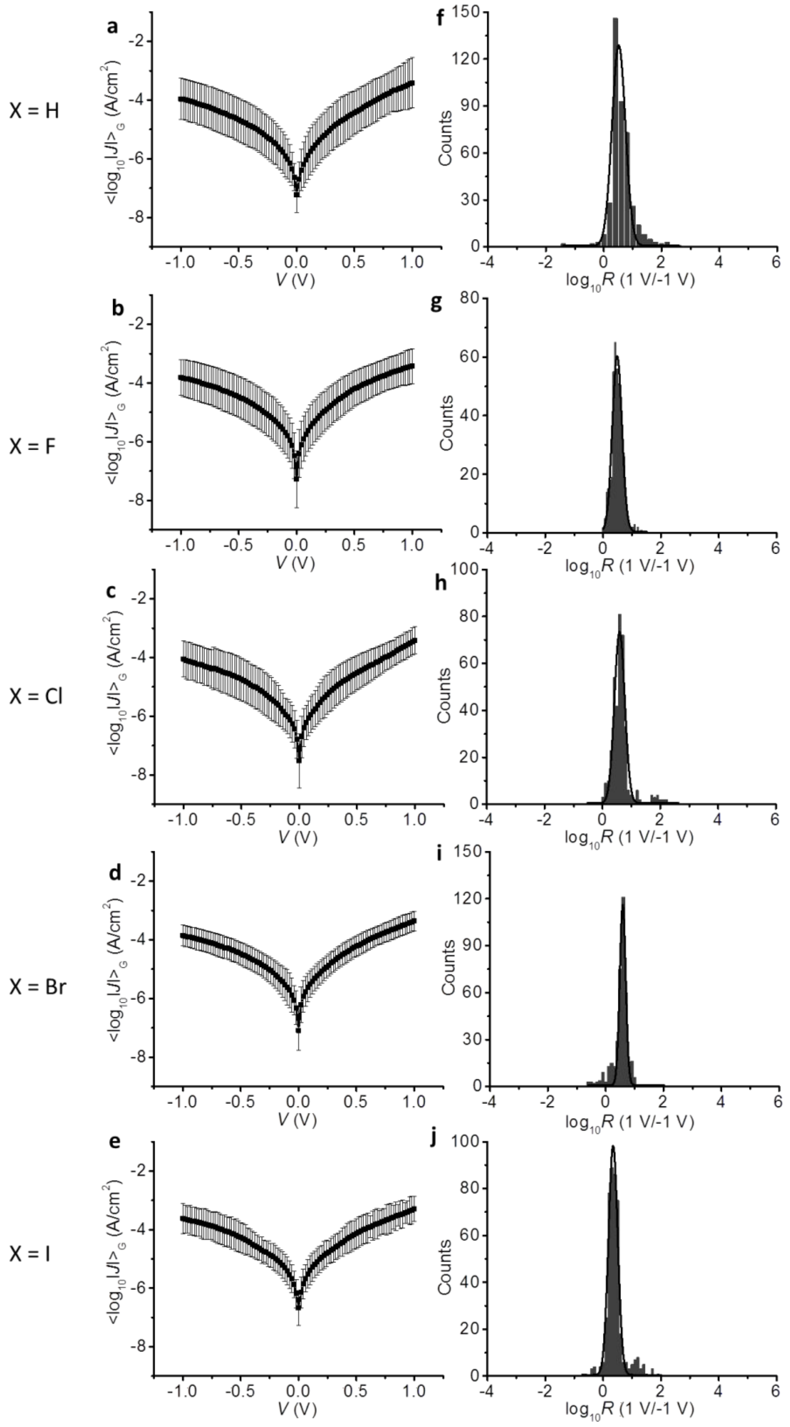

Figure S17. The $<\log_{10}|J|>_G$ vs. V(V) and histogram of $\log_{10}R$ at ±1.0 V for Ag-$S(CH_2)_{10}OPhX$//GaO_x/EGaIn junctions with X=H (a, f), F (b, g), Cl (c, h), Br (d, i), or I (e, j). The black solid symbols of the J(V) curves represent the measured $<\log_{10}|J|>_G$ at each bias, the error bars mean the $\sigma_{\log,G}$. The black solid lines in the histogram panels are the Gaussian fits.

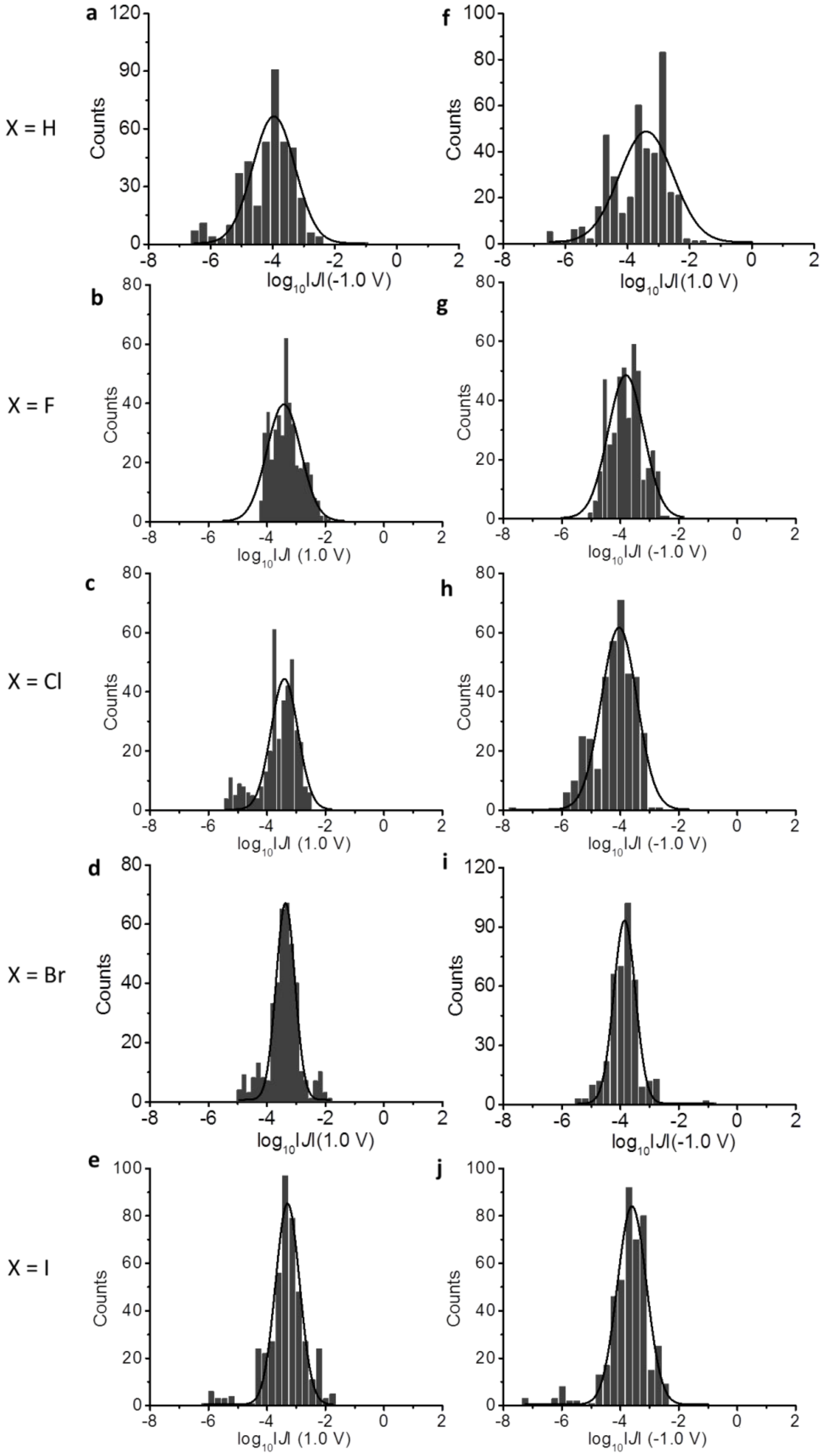

Figure S18. The histograms of $\log_{10}|J|$ at -1.0 and 1.0 V for Ag-S$(CH_2)_{10}$OPhX//GaO_x/EGaIn junctions with X=H (a, f), F (b, g), Cl (c, h), Br (d, i), or I (e, j). The black solid lines are the Gaussian fits.

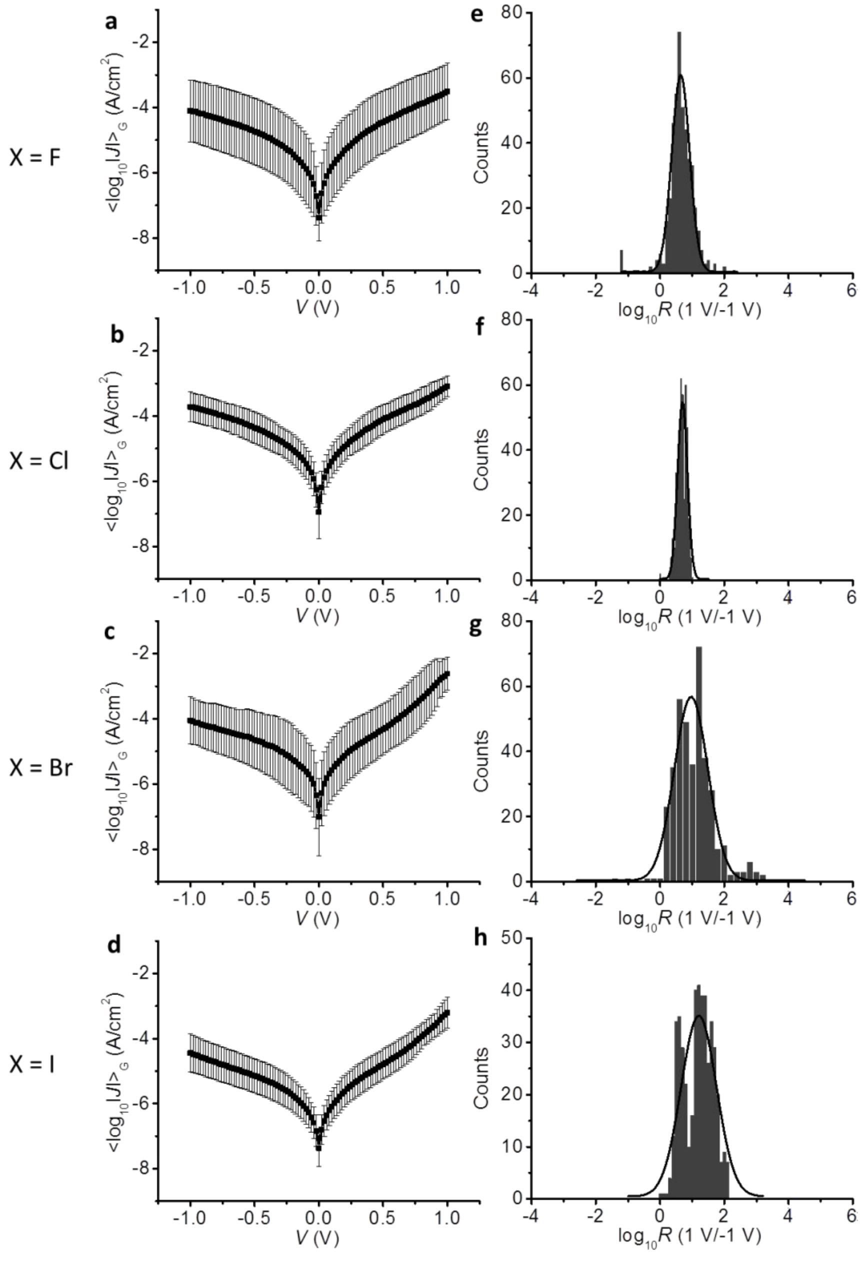

Figure S19. The $\langle \log_{10}|J|\rangle_G$ vs. V(V) and histogram of $\log_{10}R$ at ±1.0 V for Ag-S$(CH_2)_{10}$OPh$(X)_3$//GaO_x/EGaIn junctions with X=F (a, e), Cl (b, f), Br(c, g), or I (d, h). The black solid symbols of the J(V) curves represent measured $\langle \log_{10}|J|\rangle_G$ at each bias, the error bars represent the $\sigma_{\log,G}$. The black solid lines in the histogram panels are the Gaussian fits.

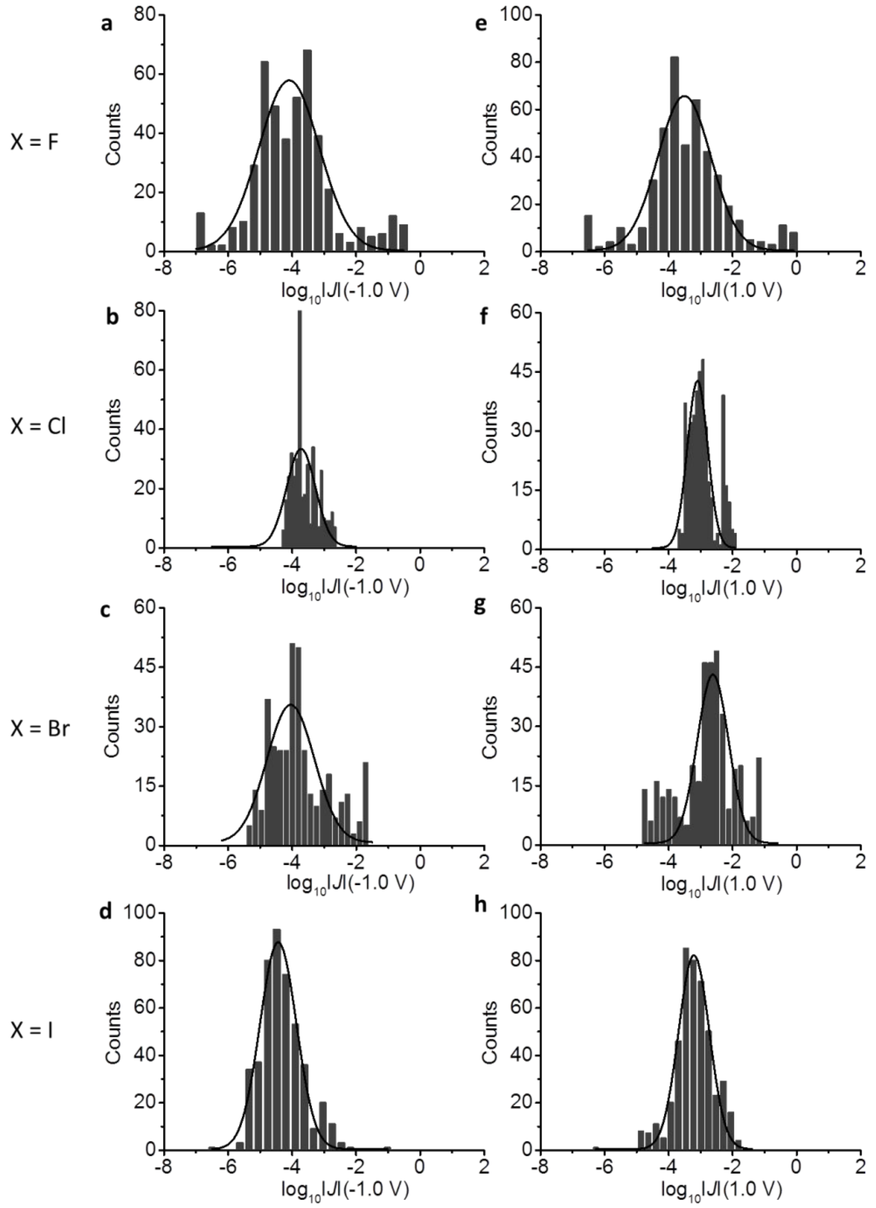

Figure S20. The histograms of $\log_{10}|J|$ at -1.0 and 1.0 V for Ag-$S(CH_2)_{10}OPh(X)_3$//GaO_x/EGaIn junctions with X=F (a, e), Cl (b, f), Br(c, g), or I (d, h). The black solid lines are the Gaussian fits.

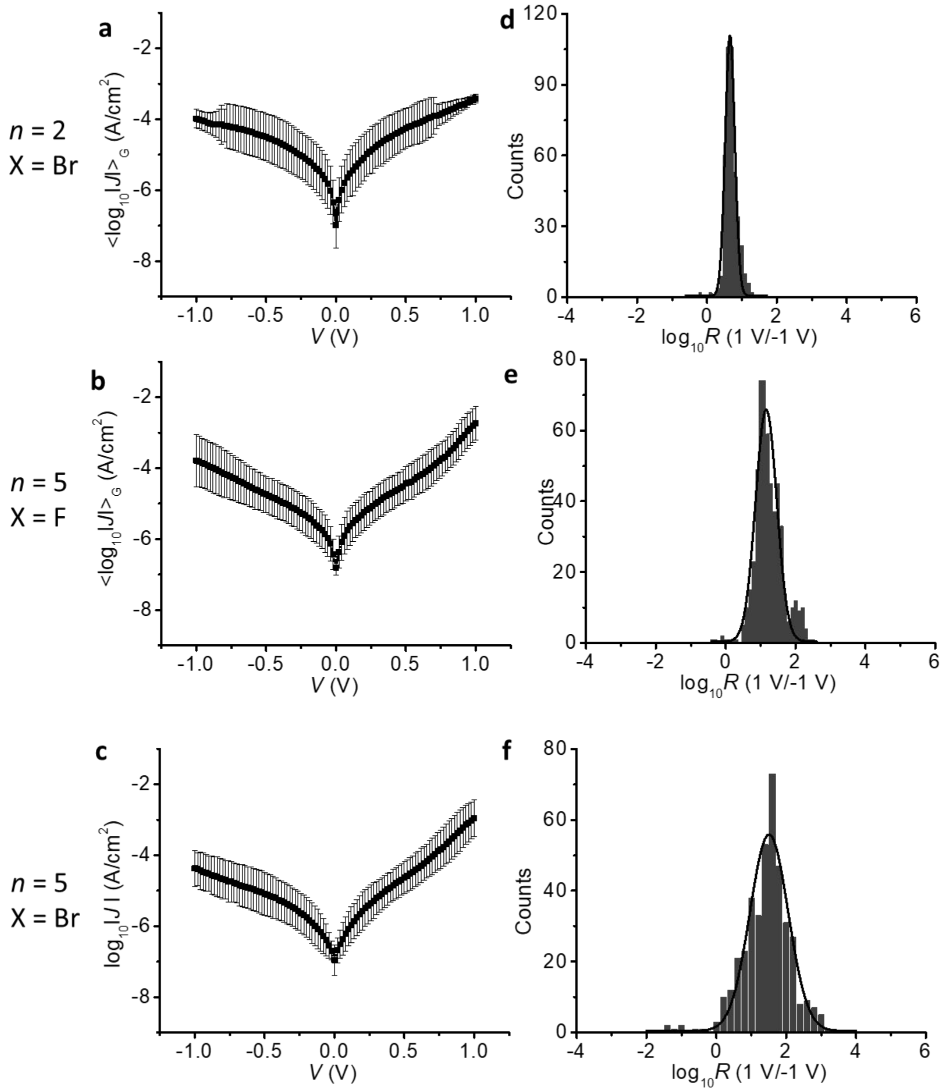

Figure S21. The $<\log_{10}|J|>_G$ vs. V(V) and histogram of $\log_{10}R$ at ±1.0 V for Ag-$S(CH_2)_{10}OPhF_n$//GaO_x/EGaIn junctions with n = 3(3,4,5-) (a, c) and n = 5 (b, d). The black solid symbols of the J(V) curves represent $<\log_{10}|J|>_G$ at measured each bias, the error bars represent$\sigma_{\log,G}$. The black solid lines in the histograms are the Gaussian fits to these histograms.

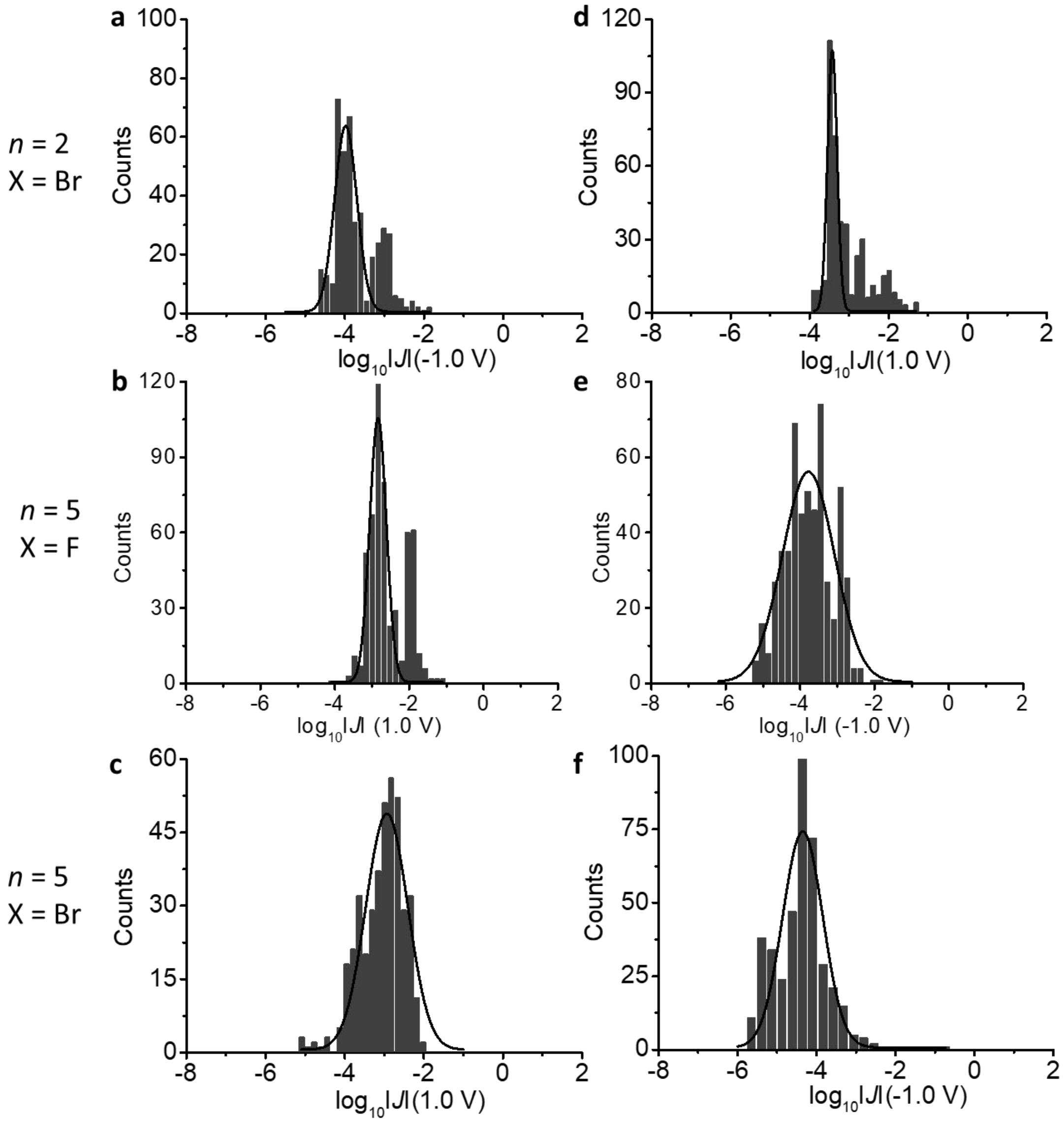

Figure S22. The histograms of $\log_{10}|J|$ at -1.0 and 1.0 V for Ag-S(CH$_2$)$_{10}$OPh(F)$_n$//GaO$_x$/EGaIn junctions with n = 3(3,4,5-) (a, c) and n = 5 (b, d). The black solid lines are the Gaussian fits.

Table S4. Summary of J(V) for junctions of Ag-S$(CH_2)_{10}$OPhX//GaO_x/EGaIn

n = 1,X =	No. of junctions[a]	No. ofshorts or unstable junctions[b]	No. of Traces[c]	Yield (%)[d]	$\langle \log_{10}\lvert J\rvert \rangle_{G(-1\,V)}(\sigma_{log,G}$[e]$)$	$\langle \log_{10}\lvert J\rvert \rangle_{G(1\,V)}(\sigma_{log,G}$[e]$)$	$\langle \log_{10}R \rangle_{G}(\sigma_{log,G})$
H	27	5	418	81	-4.0 ± 0.7	-3.4 ± 0.9	0.5 ± 0.2
F	26	4	439	85	-3.8 ± 0.6	-3.4 ± 0.6	0.5 ± 0.2
Cl	22	3	373	86	-4.1 ± 0.6	-3.4 ± 0.5	0.6 ± 0.2
Br	24	4	389	83	-3.9 ± 0.4	-3.4 ± 0.3	0.6 ± 0.2
I	26	4	436	85	-3.6 ± 0.5	-3.3 ± 0.4	0.3 ± 0.2

Table S5. Summary of J(V) for junctions of Ag-S$(CH_2)_{10}$OPhX_3//GaO_x/EGaIn

n = 3,X =	No. of junctions	No. of shorts or unstable junctions	No. of Traces	Yield (%)	$\langle \log_{10}\lvert J\rvert \rangle_{G(-1\,V)}(\sigma_{log,G})$	$\langle \log_{10}\lvert J\rvert \rangle_{G(1\,V)}(\sigma_{log,G})$	$\langle \log_{10}R \rangle_{G}(\sigma_{log,G})$
F	29	6	456	79	-4.1 ± 0.9	-3.5 ± 0.8	0.6 ± 0.3
Cl	22	1	419	95	-3.7 ± 0.4	-3.1 ± 0.3	0.7 ± 0.2
Br	26	6	380	77	-4.1± 0.7	-2.6 ± 0.5	1.0 ± 0.5
I	25	2	456	92	-4.4 ± 0.5	-3.2 ± 0.5	1.2 ± 0.5

Table S6. Summary of J(V) for junctions of Ag-S$(CH_2)_{10}$OPhF_n//GaO_x/EGaIn and Ag-S$(CH_2)_{10}$OPhBr_n//GaO_x/EGaIn.

n and X	No. of junctions	No. of shorts or unstable junctions	No. of Traces	Yield (%)	$\langle \log_{10}\lvert J\rvert \rangle_{G}$ (-1 V) $(\sigma_{log,G})$	$\langle \log_{10}\lvert J\rvert \rangle_{G}$ (1 V) $(\sigma_{log,G})$	$\langle \log_{10}R \rangle_{G}(\sigma_{log,G})$
n =5, X = F	32	5	540	84	-3.8 ± 0.7	-2.8± 0.2	1.2± 0.3
n =2, X = Br	27	5	424	81	-4.0 ± 0.3	-3.4 ± 0.1	0.7 ± 0.1
n =5, X = Br	25	4	401	84	-4.4 ± 0.5	-2.9 ± 0.5	1.6 ± 0.5

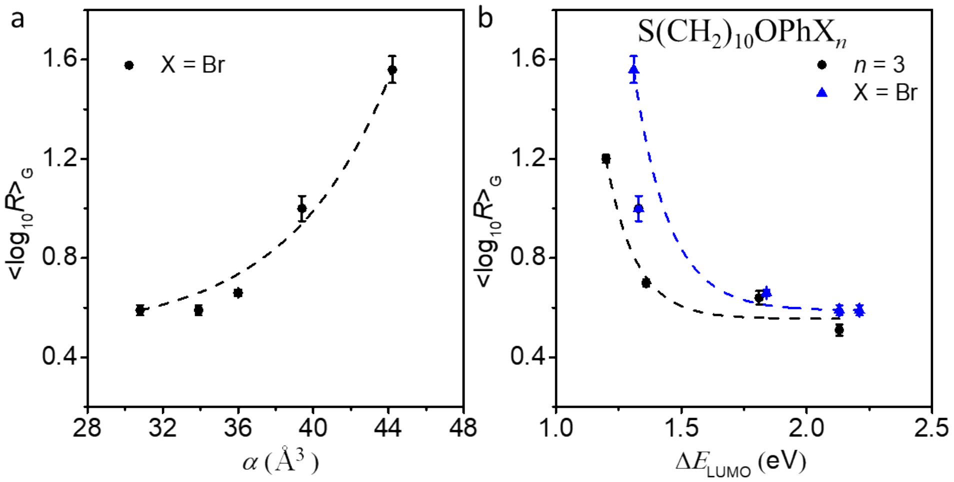

Figure S23.(a) Plot of $\langle \log_{10}R \rangle_G$ vs.α for Ag-S$(CH_2)_{10}$OPhBr_n junctions. (b) Plots of $\langle \log_{10}R \rangle_G$ vs ΔE_{LUMO} for the Ag-S$(CH_2)_{10}$OPhX_3 (n=3, black dots) and Ag-S$(CH_2)_{10}$OPhBr_n (X=Br, blue triangles) junctions.Dashed lines are guides to the eyes.

Impedance Characterization. Impedance spectroscopy is a useful technique to separate the contribution of each element like the resistance of SAM (R_{SAM} in Ω•cm^2), resistance of contact (R_C in mΩ•cm^2) and capacitance of SAM (C_{SAM} in μF/cm^2) in a circuit by applying sinusoidal voltage perturbation across a frequency range.[9-10] The corresponding ε_r can be derived from the eq 3

$$C_{SAM} = \varepsilon_0 \varepsilon_r A_{geo}/d \qquad \text{(eq S3)}$$

wherein, ε_0 is the permittivity of the free space, which is a physical constant equal to approximately 8.85×10^{-12} F/m; A_{geo} is the geometrical contact area of the junction, which is 9.616×10^{-6} cm^2; d corresponds to the thickness of the junction, here we use the SAM thickness calculated from ARXPS.The experimental setup and data analysis procedure were the same as previous report.[9] Briefly, PDMS devices confined with EGaIn in the microchannels served as top electrode.[9, 11] An impedance analyser (model Solartron 1260A with 1296A dielectric interface) was used with a standard 10pF capacitor as external reference. The sinusoidal voltage applied was 30 mV and the frequency range was 100-10^6 Hz with 10 pts/decade and 1.0s Integration. The raw impedance data obtained was fit to the equivalent circuit shown in Figure S24a. We also usedKramers–Kronig (KK) transform to check the thermostability of the junction during the sinusoidal perturbation. Figures S24-25 give the Bode Nyquist and Phase plots. The impedance results are summarized in Figure S26 and Tables S7-9.

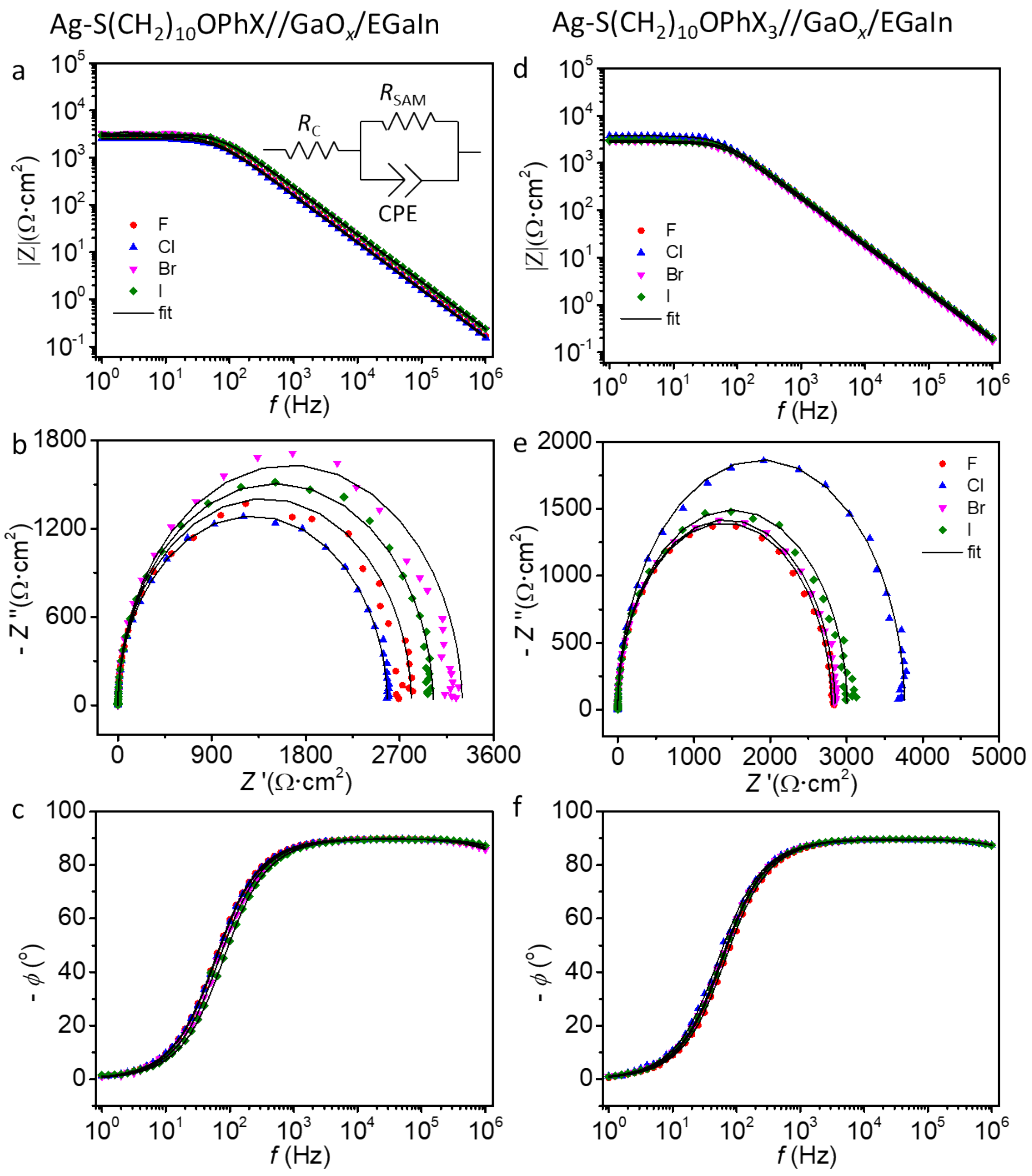

Figure S24. AC impedance measurements of the Ag-S(CH_2)$_{10}$OPhX//GaO_x/EGaIn and Ag-S(CH_2)$_{10}$OPhX_3//GaO_x/EGaIn junctions at 0 V. Frequency dependence of the modulus of the complex impedance (|Z|) for different types of molecular junctions (a and d); the corresponding Nyquist plots (b and e) and phase angle vs. frequency plots (c and f).

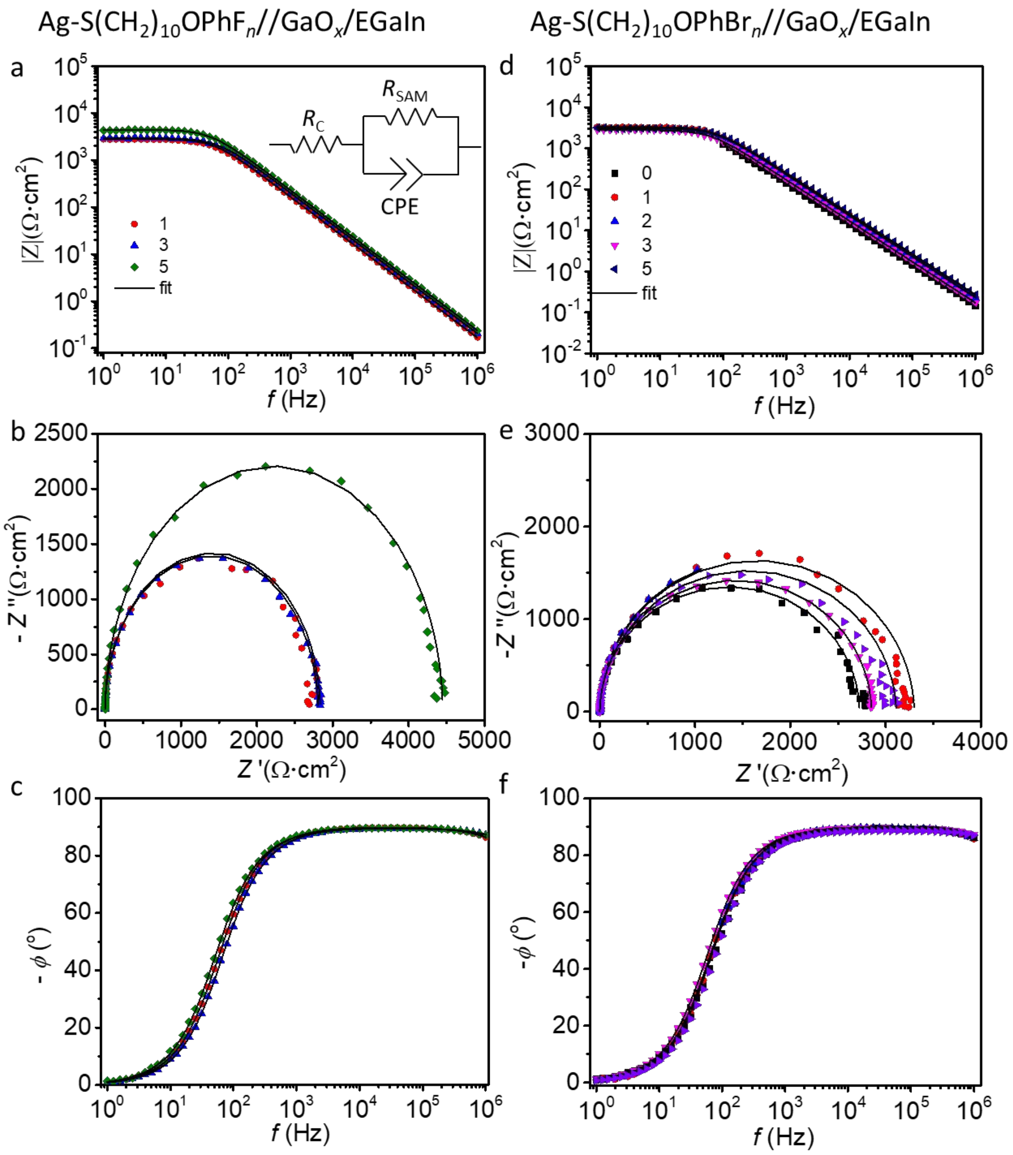

Figure S25. AC impedance measurements of the Ag-S(CH_2)$_{10}$OPhF$_n$//GaO$_x$/EGaIn and Ag-S(CH_2)$_{10}$OPhBr$_n$//GaO$_x$/EGaIn junctions at 0 V. Frequency dependence of the modulus of the complex impedance (|Z|) for different types of molecular junctions (a and d); the corresponding Nyquist plots (b and e) and phase angle vs. frequency plots (c and f).

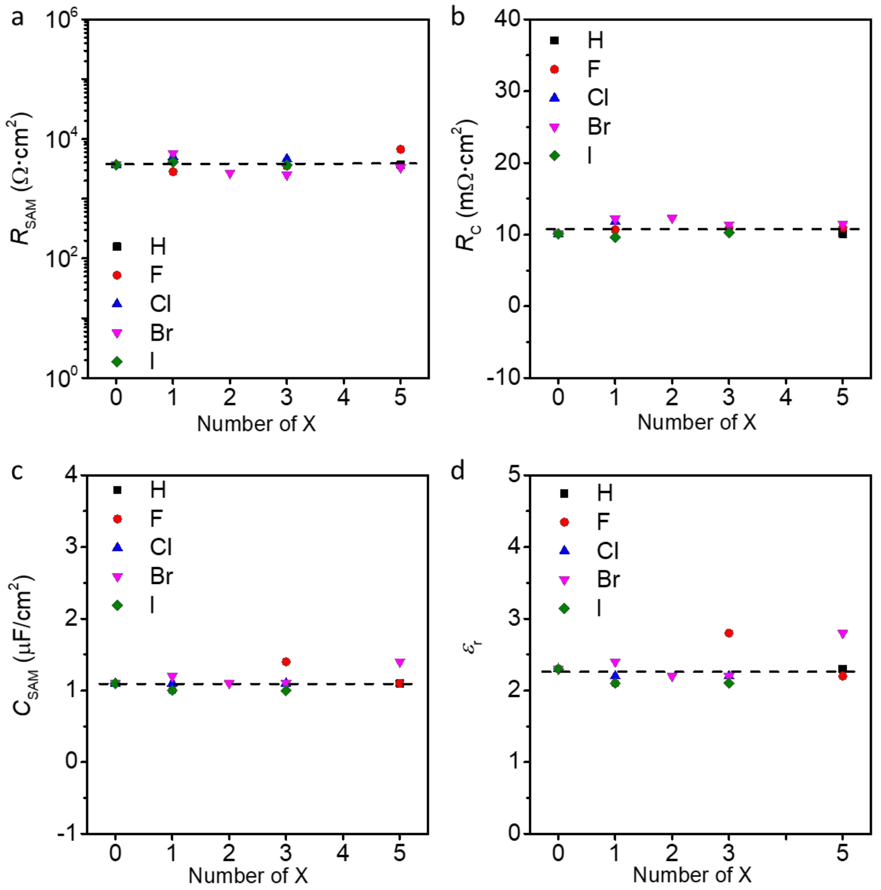

Figure S26. Averaged R_{SAM} (**a**), R_C (**b**), C_{SAM} (**c**),and ε_r (**d**) of Ag-S$(CH_2)_{10}$OPhX_n//GaO_x/EGaIn junctions at 0 V from impedance spectroscopies. Error bar shown in Tables S7-9. The black dashed lines are guides to the eyes.

Table S7. Summary of impedance results of junctions of Ag-S$(CH_2)_{10}$OPhX//GaO_x/EGaIn.

n = 1, X =	R_{SAM}(kΩ•cm^2)	R_C (mΩ•cm^2)	C_{SAM} (μF/cm^2)	ε_r	n_{CPE}
H	4±2	10±2	1.1±0.5	2.3±0.9	0.994
F	3±1	11±1	1.0±0.2	2.1±0.4	0.995
Cl	5±4	12±2	1.1±0.2	2.2±0.3	0.995
Br	6±2	12±3	1.2±0.4	2.4±0.8	0.998
I	4±2	10±2	1.0±0.3	2.1±0.6	0.997

Table S8. Summary of impedance results of junctions of Ag-S$(CH_2)_{10}$OPhX_3//GaO_x/EGaIn.

n = 3, X =	R_{SAM} (kΩ•cm^2)	R_C (mΩ•cm^2)	C_{SAM} (μF/cm^2)	ε_r	n_{CPE}
F	4±2	11±2	1.4±0.3	2.8±0.7	0.996
Cl	5±2	11±2	1.1±0.1	2.2±0.2	0.998
Br	3±1	11±3	1.1±0.1	2.2±0.2	0.995
I	4±1	10±2	1.0±0.1	2.1±0.2	0.996

Table S9. Summary of impedance results of junctions of Ag-S$(CH_2)_{10}$OPhX$_n$//GaO$_x$/EGaIn.

$n$and X	R_{SAM} (kΩ•cm^2)	R_C (mΩ•cm^2)	C_{SAM} (μF/cm^2)	ε_r	n_{CPE}
n =5, X = F	7±3	11±3	1.1±0.3	2.2±0.7	0.989
n =2, X = Br	3±1	12±2	1.1±0.2	2.2±0.3	0.995
n =5, X = Br	3±3	11±2	1.4±0.1	2.8±0.2	0.994
n =5, X = Br	3±2	5±1	6.2±1.0	13±2	0.956

***J*(V, T) measurementsand data analysis.** The *J*(V,T) measurements were conducted in a probe station (Lakeshore CRX-VF) connected to a chiller (Shelton Chillers, SAE-AC5, SHELTON(S) PTE LTD) and compressor (Sumitomo CRYOGENICS, F-50, Sumitomo Heavy Industries. Ltd.) at a pressure of 5×10^{-5} bar. For *J*(V, T) measurement, PDMS (polydimethylsiloxane) devices with microfluidic channels were used instead of EGaIn conical tip.[11] Before *J*(V,T) data collection, we checked the *J*(V) of the junction to make sure the current density $\log_{10}|J|$was within one log-standard deviation of the conical tip results. We collected *J*(V) in the temperature range of 250-340 K at an interval of 10 K and at each temperature 10 traces were collected and averaged, which were used for the Arrhenius plot analysis and the activation energy (E_a) calculation. For each type of SAM, we measured 3 junctions to average the E_a and to obtain the standard deviation. Figures S27-29 show the *J*(V,T) curves and the corresponding Arrhenius plots for the junctions.

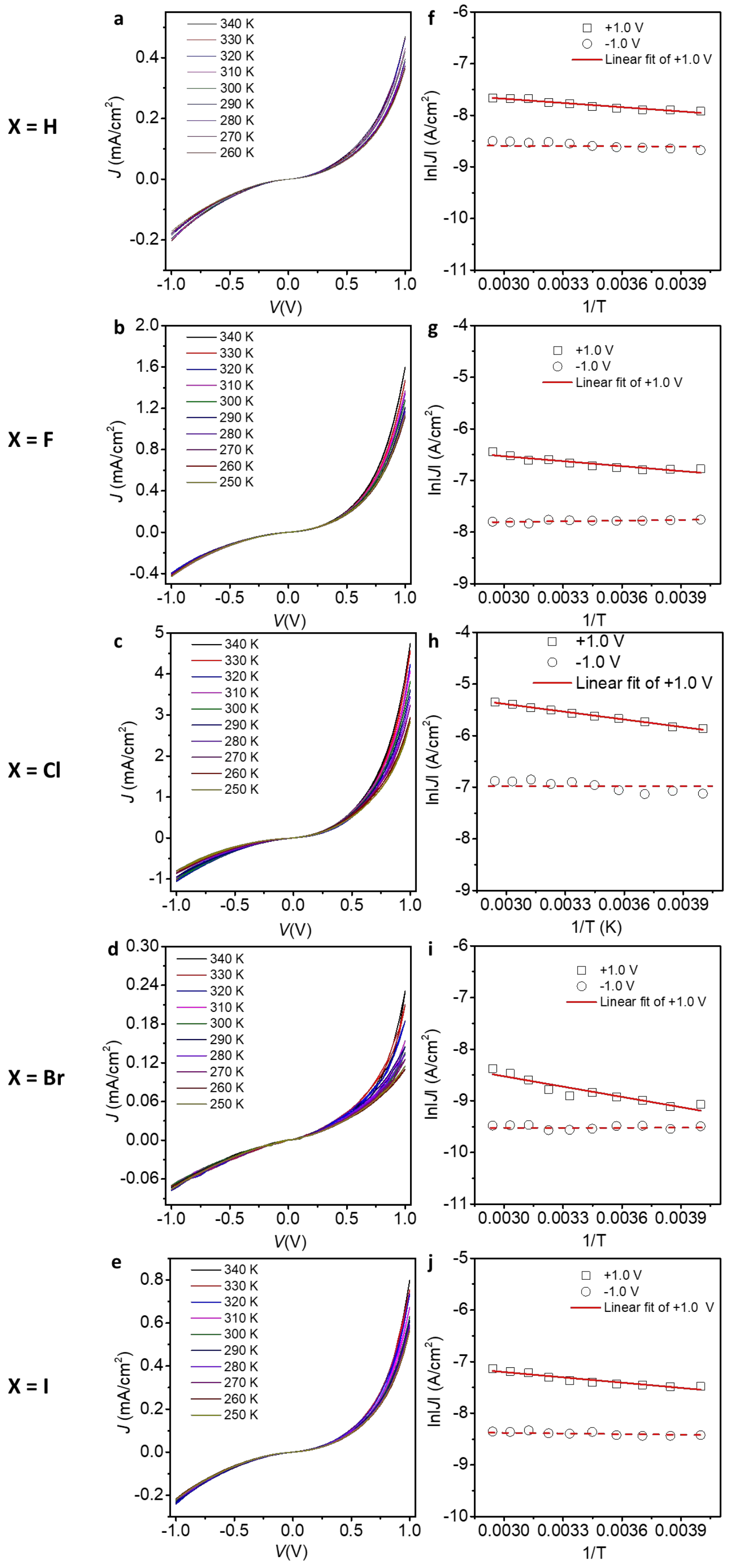

Figure S27. (a-e)J(V) of Ag-S(CH_2)$_{10}$OPhX//GaO_x/EGaIn under temperature range of 250 – 340 K. (f-j) ln|J| at +1.0 V and -1.0 V as a function of 1/T. Red solid lines are the linear fits, the red dash lines are guides to the eyes.

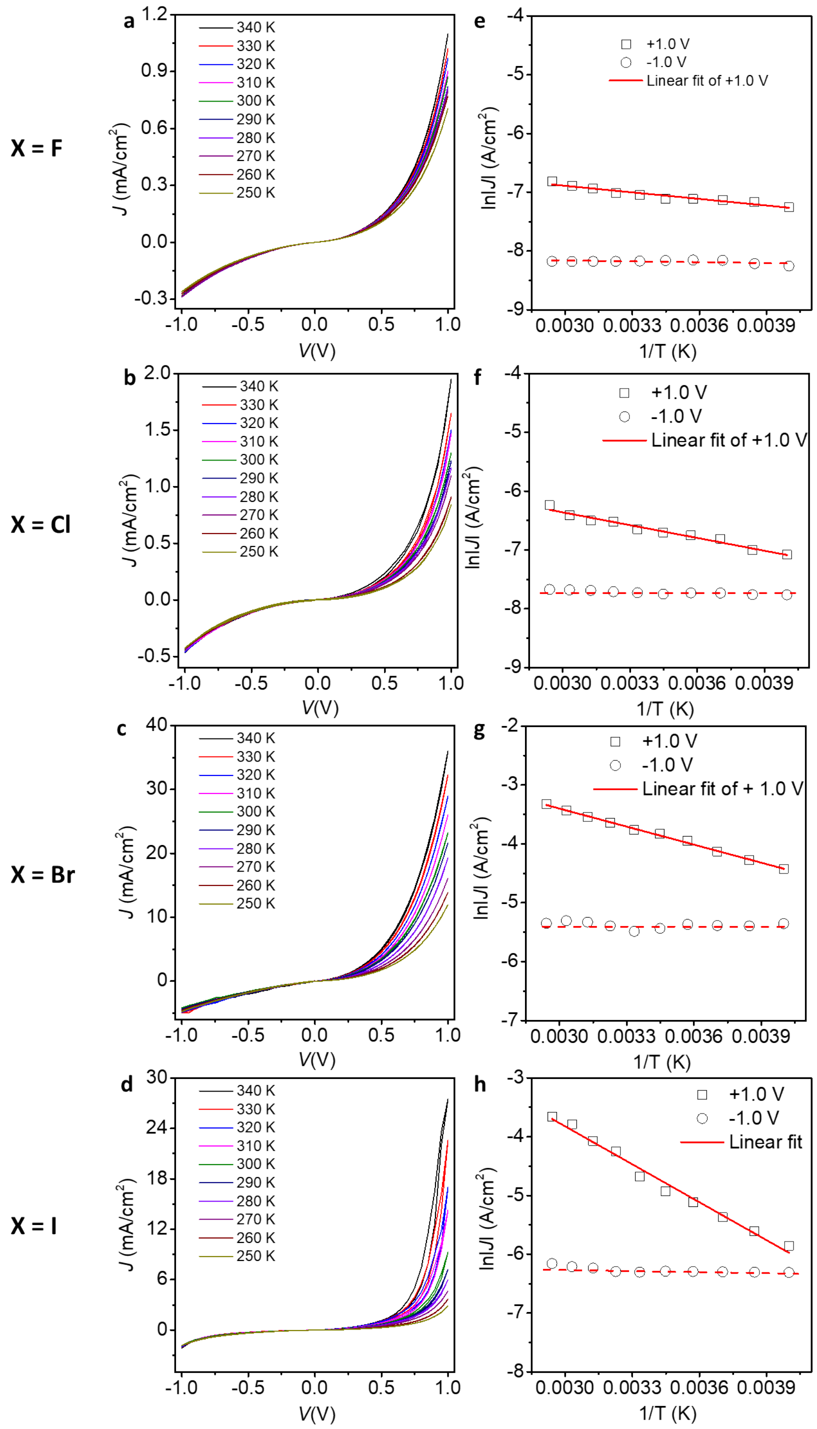

Figure S28. (a-d)J(V) of Ag-S(CH$_2$)$_{10}$OPhX$_3$//GaO$_x$/EGaIn under temperature range of 250 – 340 K. (e-h)ln|J| at +1.0 V and -1.0 V as a function of 1/T. Red solid lines are the linear fits, the red dash lines are guides to the eyes.

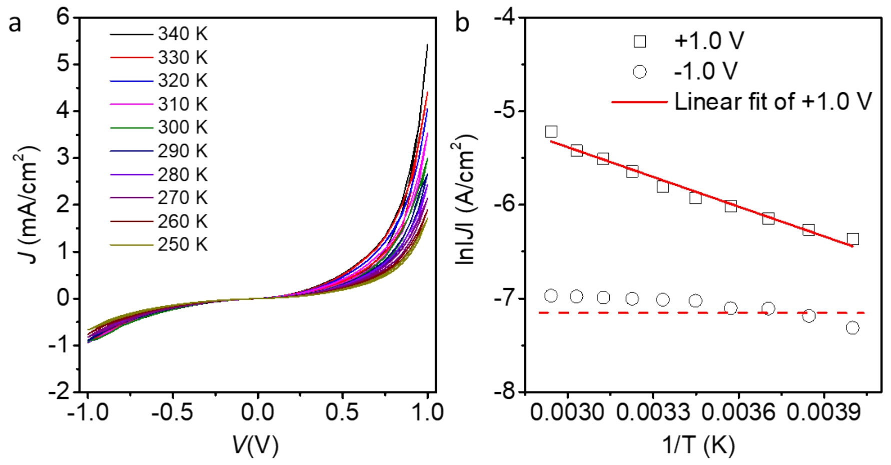

Figure S29. (a)J(V) of Ag-S$(CH_2)_{10}$OPhF_5//GaO_x/EGaIn under temperature range of 250 – 340 K. (b)ln|J| at +1.0 V and -1.0 V as a function of 1/T. Red solid lines are the linear fit to the ln|J| at +1.0 V, the red dash line is a guide to the eyes.

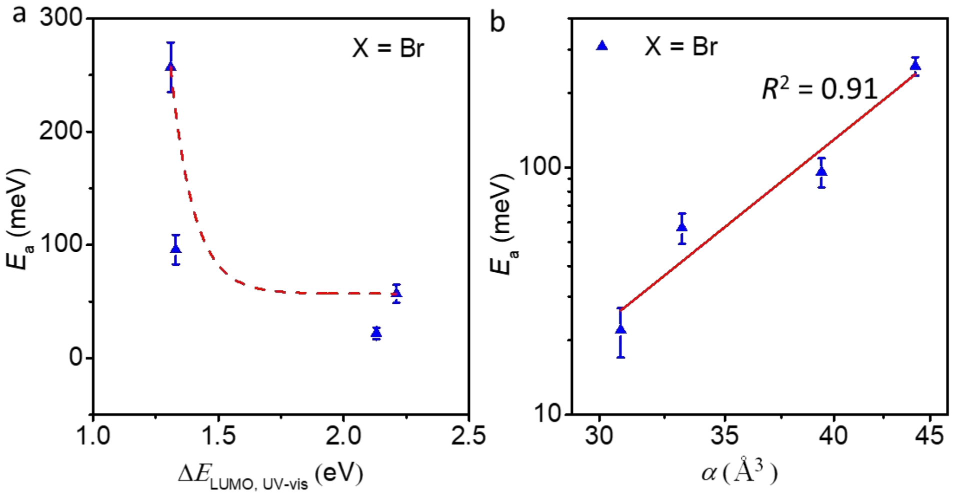

Figure S30. Plots of E_a vs ΔE_{LUMO} (a) andE_a vs α (b) for junctions of Ag-S$(CH_2)_{10}$OPhBr_n//GaO_x/EGaIn. The error bars are the standard deviation from three measurements. The dashed line is guide to the eye. The solid line is linear fit of the plot.

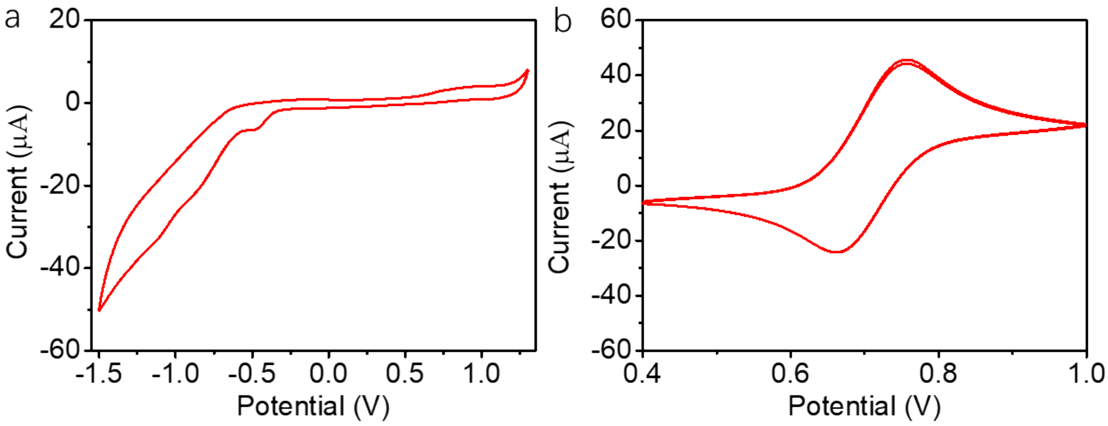

Figure S31. (a) Cyclic voltammogram of 5 mM $BrS(CH_2)_{10}OPhBr_5$ in 0.1 M tetrabutylammonium hexafluorophosphate in acetonitrile solution (degassed using argon for 30 min) in the potential range of -1.5 to 1.3 V (the inert potential range of Au electrode)[12]. (b) 5 mM ferrocene in the same electrolyte was used as control. The working electrode was Au, and Pt and Ag wires were used as counter and reference electrodes, respectively. The scan rate was 0.1 V/s. The small peak at -0.5 V is the reduction peak of residual oxygen.[13]

Table S10. Summary of E_a for junctions of Ag-$S(CH_2)_{10}OPhX_n$//GaO_x/EGaIn. The error bar indicates the standard deviation from three different measurements.

n and X	E_a (meV)
n = 1, X = H	22 ± 5
n = 1, X = F	28 ± 4
n = 1, X = Cl	43 ± 11
n = 1, X = Br	57 ± 8
n = 1, X = I	30 ± 4
n = 3, X = F	35 ± 6
n = 3, X = Cl	62 ± 10
n = 3, X = Br	96 ± 13
n = 3, X = I	176 ± 21
n = 5, X = F	92 ± 9
n = 5, X = Br	257 ± 22

References

1. Yamaguchi, I.; Goto, K.; Sato, M., Enzyme and transition-metal-complex catalyzed synthesis of polyphenols with pendant oligo(p-phenylene) and their optical, electrochemical, and thermal properties. *Macromolecules* **2009,** *42*, 7836-7845.

2. Nerngchamnong, N.; Yuan, L.; Qi, D.-C.; Li, J.; Thompson, D.; Nijhuis, C. A., The role of van der Waals forces in the performance of molecular diodes. *Nat. Nanotechnol.* **2013,** *8*, 113-118.
3. Holmes, B. T.; Snow, A. W., Aliphatic thioacetate deprotection using catalytic tetrabutylammonium cyanide. *Tetrahedron* **2005,** *61*, 12339-12342.
4. Chen, X.; Roemer, M.; Yuan, L.; Du, W.; Thompson, D.; Del Barco, E.; Nijhuis, C. A., Molecular diodes with rectification ratios exceeding 10(5) driven by electrostatic interactions. *Nat. Nanotechnol.* **2017,** *12*, 797-803.
5. Yu, X.; Wilhelmi, O.; Moser, H. O.; Vidyaraj, S. V.; Gao, X.; Wee, A. T. S.; Nyunt, T.; Qian, H.; Zheng, H., New soft X-ray facility SINS for surface and nanoscale science at SSLS. *J. Electron Spectrosc. Relat. Phenom.* **2005,** *144-147*, 1031-1034.
6. Yuan, L.; Breuer, R.; Jiang, L.; Schmittel, M.; Nijhuis, C. A., A molecular diode with a statistically robust rectification ratio of three orders of magnitude. *Nano Lett.* **2015,** *15*, 5506-5512.
7. Makuła, P.; Pacia, M.; Macyk, W., How to correctly determine the band gap energy of modified semiconductor photocatalysts based on UV–Vis spectra. *J. Phys. Chem. Lett.* **2018,** *9*, 6814-6817.
8. Chiechi, R. C.; Weiss, E. A.; Dickey, M. D.; Whitesides, G. M., Eutectic gallium–indium (EGaIn): a moldable liquid metal for electrical characterization of self-assembled monolayers. *Angew. Chem. Int. Ed.* **2008,** *47*, 142-144.
9. Sangeeth, C. S. S.; Wan, A.; Nijhuis, C. A., Equivalent circuits of a self-assembled monolayer-based tunnel junction determined by impedance spectroscopy. *J. Am. Chem. Soc.* **2014,** *136*, 11134-11144.
10. Chen, X.; Nijhuis, C. A., The unusual dielectric response of large area molecular tunnel junctions probed with impedance spectroscopy. *Adv. Electron. Mater.* **2022,** *8*, 2100495.
11. Wan, A.; Jiang, L.; Sangeeth, C. S. S.; Nijhuis, C. A., Reversible soft top-contacts to yield molecular junctions with precise and reproducible electrical characteristics. *Adv. Funct. Mater.* **2014,** *24*, 4442-4456.
12. Laura, C.; Galyna, S.; Daniel, B., Electrochemical behavior of platinum, gold and glassy carbon electrodes in water-in-salt electrolyte. *Electrochem. Commun.* **2017**, *77*, 89-92.
13. Ma, Z., Dou, S., Shen, A., Tao, L., Dai, L. Wang, S. Sulfur-doped graphene derived from cycled lithium–sulfur batteries as a metal-free electrocatalyst for the oxygen reduction reaction. *Angew. Chem. Int. Ed.*, **2015**, *54*, 1888-1892.
